# Mediators of outcome in adolescent psychotherapy and their implications for theories and mechanisms of change: a systematic review

**DOI:** 10.1007/s00787-023-02186-9

**Published:** 2023-03-15

**Authors:** Svenja Taubner, Yianna Ioannou, Andrea Saliba, Célia M. D. Sales, Jana Volkert, Sonja Protić, Asta Adler, Rasa Barkauskiene, Sonia Conejo-Cerón, Dina Di Giacomo, Jose M. Mestre, Patricia Moreno-Peral, Filipa Mucha Vieira, Catarina Pinheiro Mota, Margarida Isabel Rangel Santos Henriques, Jan Ivar Røssberg, Tjasa Stepisnik Perdih, Stefanie Julia Schmidt, Max Zettl, Randi Ulberg, Erkki Heinonen

**Affiliations:** 1 Institute for Psychosocial Prevention, University Heidelberg, Bergheimer Str. 52, 69115 Heidelberg, Germany; 2 University of Nicosia, Nicosia, Cyprus; 3 University of Malta and Mental Health Services Malta, Msida, Malta; 4 Faculty of Psychology and Education Science, University of Porto, Porto, Portugal; 5 Center for Psychology, University of Porto, Porto, Portugal; 6 Department of Psychology, Medical School Berlin, Berlin, Germany; 7 Institute of Criminological and Sociological Research Belgrade, Belgrade, Serbia; 8 Institute of Psychology, Vilnius University, Vilnius, Lithuania; 9 Instituto de Investigación Biomédica de Malaga, Málaga, Spain; 10 Life, Health and Environmental Sciences, University of L’Aquila, L’Aquila, Italia; 11 Universidad de Cadiz, Cadiz, Spain; 12 University of Trás-Os-Montes and Alto Douro, Vila Real, Portugal; 13 Institute of Clinical Medicine, University of Oslo, Oslo, Norway; 14 Oslo University Hospital, Oslo, Norway; 15 School of Advanced Social Studies, Nova Gorica, Slovenia; 16 Department of Clinical Child and Adolescent Psychology, University of Bern, Bern, Switzerland; 17 National Institute for Health and Welfare, Helsinki, Finland; 18 Diakonhjemmet hospital, Oslo, Norway; 19 International Psychoanalytic University, Berlin, Germany

**Keywords:** Change mechanism, Mediator, Review, Adolescence, Psychotherapy, RCT

## Abstract

Psychotherapeutic treatment of adolescents requires age-specific approaches and thus plausibly also involves different change mechanisms than adult psychotherapy. To guide further research and improve therapeutic outcomes for adolescents, we reviewed all RCTs investigating mechanisms of change in the psychological treatment of adolescents to identify the most promising age-, disorder- or treatment-specific mediators. Following the preferred reporting items for systematic reviews (PRISMA), 106 studies were included that reported 252 statistical mediation tests assessed with 181 different measures. Most often studied and significant mediators were cognitive, followed by family-related, and behavioral variables. Several mediators were identified to be promising for future investigations: changes in negative thoughts, dysfunctional beliefs and metacognitive skills; family functioning and parenting skills; as well as successful engagement in therapy activities and increased impulse control. Symptom change during therapy was least often a mediator for other therapeutic changes. Relational and emotional mediators were largely understudied, whereas peer-influence appeared a promising mediator for intervention outcomes. Adolescence-specific mediators were most commonly investigated. Majority of studied mediators were not disorder-specific. There was a tendency to mainly test change mechanisms of specific theoretical models without considering other possible change theories. Further, virtually no studies fulfilled all criteria for rigorously investigating mediation and only nine were classified with an overall good study quality. While bearing in mind the current limitations in study designs, methodological rigor and reporting, there appears to be substantial evidence for transdiagnostic age-specific change models in the psychological treatment of adolescents. For future research, need for consensus on a core set of transdiagnostic and transtheoretical mediators and measures is highlighted. These should address likely core mechanisms of change, as well as take into account age-relevant developmental challenges and biological markers.

**Supplementary Information:**

The online version contains supplementary material available at 10.1007/s00787-023-02186-9.

## Introduction

Adolescents are a particularly important and vulnerable group with distinct mental health needs due to the developmental changes in this age period. According to Kessler and colleagues, 50% of lifetime diagnosable mental health disorders start before the age of 14, and this number increases to 75% before the age of 25 [1]. Ignoring young people's mental health needs can result in long-lasting adverse developmental outcomes for the individual and society. These include mental disorders in adulthood, impaired social and role functioning, difficulties with employment, as well as poorer general health outcomes [2]. In contrast, helping adolescents overcome mental health problems lays the foundation for their adult health and the health of their offspring. Thus, addressing adolescent mental health is of utmost importance.

Psychological treatment is one of the important keys for the promotion of youth mental health, given the extensive evidence on the general effectiveness of psychotherapy for treating mental disorders in adolescence [3, 4]. However, effect sizes are smaller when treating adolescents as opposed to adults, especially in the case of multiple simultaneous problems (e.g., comorbidity and social problems) [5]. This likely reflects at least partly the fact that psychotherapy with adolescents differs from the treatment of adults in multiple regards [6]. For example, in contrast to adults, adolescents are more often not self-referred but rather sent to therapy by others and may, therefore, be less intrinsically motivated for treatment. They may also present with age-specific symptoms, diagnoses, and target complaints [7]; their treatment more likely involves third parties (such as guardians, teachers, social workers, etc.); and they more likely have fewer shared interests and differ more in both age and value-systems with their therapists; all of which can compromise agreeing on therapy tasks and goals and achieving a good therapeutic alliance and, subsequently, outcomes [8, 9]. 

Furthermore, with more than 550 different posited psychotherapy models, potentially also applicable to young people, little apparent consensus exists on how psychotherapy works [10]. Thus, to increase and optimize treatment outcomes for young people, processes that facilitate successful therapeutic change should be empirically identified irrespective of therapeutic schools. By identifying transtheoretical treatment processes that facilitate successful therapeutic change and isolating those that are redundant and can be dismissed, it becomes possible to optimize treatment outcomes for this age group. This review summarizes all studies that have investigated mechanisms of change in the psychological treatment of adolescents by analyzing change mechanisms in relation to outcome in randomized controlled trials (RCTs).

An important first step towards examining mechanisms of change in psychotherapy is the identification of mediators [11]. While patient-related moderators (e.g., gender, age) serve to clarify what kind of treatment works for which kind of person, mechanisms of change define causal relationships between therapeutic change and psychological interventions. A mechanism of change explains how an intervention translates into a process that leads to an outcome, e.g., change in symptoms [12]. Thus, a mechanism is an explanatory concept that relies on identifying mediators, i.e., variables that explain changes between an intervention and one or more therapeutic outcomes statistically. Kazdin has formulated clear criteria on how to assess mediators of psychological treatments [13] as follows:

- Conduct sufficiently powered randomized clinical trials
- Use valid and reliable measures for mediators that are sensitive to change
- Apply a process design in which changes of the mediator temporally precede changes in therapeutic outcome and the mediator variable is measured repeatedly
- Compare mediators that are theory-driven with non-specific mediators
- Apply different dosages to prove that a stronger mediator-change leads to more therapeutic changes

After 16 years of Kazdin’s suggestions on how to assess mechanisms of change in psychotherapy research, the extant lack of knowledge remains striking. In their review, Cuijpers et al. [14] concluded that despite more than 70 years of systematic empirical psychotherapy research, we still have no empirically validated mechanisms of change in psychotherapy, neither in terms of common nor specific factors (i.e., mechanisms operating across or solely within particular therapeutic approaches)—yet the evidence base is even smaller in psychotherapy for children and adolescents. However, there has been an increasing number of publications on mechanisms of change and recent attempts to synthesize the empirical findings. We have found 20 newer systematic reviews and/ or meta-analyses investigating change mechanisms in psychotherapy which used Kazdin’s criteria when discussing the robustness of the evidence. The majority of these reviews investigated studies with adults and focused on one specific change mechanism in one specific treatment model for one specific disorder, e.g., repetitive negative thinking in CBT for depression [15]; threat reappraisal [16], cognitive changes [17], or panic efficacy [18] in CBT for anxiety disorders; as well as mindfulness-based interventions for the treatment of depressive rumination [19]. Other reviews investigated several mediators with one specific therapy model related to one specific disorder, e.g., CBT for drug use disorder [20], CBT for insomnia [21], and CBT for irritable bowel syndrome [22]. Yet others examined proof for theory-driven change mechanisms in specific therapeutic approaches, e.g., Mindfulness-Based Cognitive Therapy [23], Motivational Interviewing [24] or Acceptance and Commitment Therapy [25]. A further category of reviews summarized evidence related to specific diagnoses across different therapeutic approaches, e.g., mechanisms in Internet- and mobile-based intervention for adult depression [26] or adult PTSD [27], or treatments for depression in adults, children and adolescents [28], and prevention of depression and anxiety [29], also across all ages. In addition, one systematic review and meta-analysis was dedicated to CBT for the treatment of anxiety in children and adolescents [30], one systematic review summarized studies on change mechanisms in psychological treatments for depressed adolescents [31], and another was dedicated to change mechanisms in externalizing disorders in adolescents and children [32]. A recent scoping review took a more comprehensive approach and examined predictors, moderators, and mediators associated with treatment outcome in randomized clinical trials among adolescents with depression [33]. Last, some reviews focused on specific mediators irrespective of diagnoses or treatment approaches, such as alliance [34] and insight [35]. Table 1 summarizes the reviews of studies that focused exclusively on adults and below, we will summarize only reviews of studies that included children and adolescents.

**Table 1:** Summary of reviews and meta-analysis in adult psychotherapy

Author	Topic	Main findings
Specific mediators for specific diagnoses in specific therapies		
Spinhoven et al. [15]	Changes in repetitive negative thinking for the treatment of depression in CBT (Meta-Analysis)	36 RCTs but direction of the effect and causality remained unclear
Smits et al. [16]	Changes in threat reappraisal for the treatment of anxiety disorders in CBT (Meta-Analysis)	25 RCTs with inconclusive results regarding treatment differences, suggesting that the mechanism is not specific to CBT, causality not established
Breuninger et al. [17]	Changes in cognitions in CBT for anxiety disorders (Review)	Of 30 RCTs, 17 studies supported the change theory, however changes in positive cognitions such as self-efficacy had stronger effects, contradicting or expanding CBT change theories
Fentz et al. [18]	Changes in panic self-efficacy and catastrophic beliefs in CBT for panic disorder (Review)	Of 33 original studies, only three carried out statistical mediation and only one yielded statistically significant results and the effects were not specific for CBT
Perestelo-Perez et al. [19]	Changes in rumination in depression using mindfulness-based therapies (Review)	Five out of 11 studies investigated the mediating effect of increased mindfulness/ acceptance and yielded inconclusive results
Several mediators for specific diagnoses in specific therapies		
Magill et al. [20]	Change mechanisms in CBT in alcohol and drug abuse disorders (Meta-Analysis)	Coping and self-efficacy were the strongest mediators in ten studies, but it remained unclear if they were related to a CBT-specific change process
Radu et al. [22]	Changes in CBT for irritable bowel syndrome (Meta-Analysis)	Six studies with cognitive, emotional and behavioral mediators, with cognitive explaining the smallest amount of variance
Parsons et al. [21]	Internet-based CBT (CBT-I) on sleep-related problems (Meta-Analysis)	11 RCTs found some evidence for cognitive mediators and no evidence for behavioral mediators
Therapy-specific mediators		
Gu et al. [23]	Change mechanisms for mindfulness-based therapies (Meta-Analysis)	Changes in mindfulness (from 12 RCTs) and repetitive negative thinking (from six RCTs), but findings derived from a secondary analysis and not from the original studies. Inconclusive results for the effect of changes in self-compassion and psychological flexibility
Romano and Peters [24]	Change mechanisms in Motivational Interviewing (Meta-Analysis)	19 studies showed no effect on patients’ motivation; significant mediation effects emerged in six studies with regard to in-session engagement (mainly measured as working alliance)
Stockton et al. [25]	Six change mechanisms in Acceptance and Commitment Therapy (Review)	Only six of the 12 included studies used formal mediation analysis and showed robust evidence only for acceptance
Diagnosis-specific mediators		
Domhardt et al. [26]	Internet-based Interventions for depression (Review)	64 different mediators, mainly CBT trials and cognitive mediators, considerably less studied mediator groups were behavioral and emotional mediators
Steubl et al. [27]	Internet-based Interventions for the treatment of PTSD (Review)	Three trials with four different and significant mediators: self-efficacy beliefs, perceived physical impairment, social acknowledgement, and disclosure of trauma
Isolated mediators		
Baier et al. [34]	Therapeutic alliance as a putative mechanism of change (transdiagnostic) (Review)	37 RCTs, of which about half were CBT, the majority of the studies (70%) found evidence for a mediating role of alliance on outcome. However, there was heterogeneity in the quality of studies and only seven were considered high quality
Jennissen et al. [35]	Insight as a curative transtheoretical factor (Review)	22 studies (majority psychodynamic therapy), concluding that insight could be a relevant mechanism of change across different therapeutic schools

### Diagnosis-specific mediators in children and adolescents

For the non-adult reviews on mediators only diagnosis-specific mediators have been investigated so far. Lemmens et al. [28] summarized a variety of 39 potential psychological mediators in the treatment of depression across all age groups using 35 original studies of mainly CBT (21 studies), mindfulness-based interventions (five studies), and several other treatments. Due to the dominance of CBT studies, mediators were predominantly cognitive (negative thoughts, dysfunctional attitudes, attributional style), while others were quite closely cognition-related, such as mindfulness, worry, and rumination; and a few related to behavior and alliance. Half of the studies found evidence for mediation and the other half did not. 80% of the studies included more than one mediator. However, these mediators were tested mainly individually and rarely tested for how much incremental variance in effectiveness they explained. Only 12 studies fulfilled the temporal criterion for mediation testing and no study fulfilled all of Kazdin’s criteria. Studies with a non-active control group had more significant mediators, which may point to the interpretation that the mediators tested in these studies were not specific to a certain approach. Moreno-Peral et al. [29] reported on 26 RCTs for the prevention of depression and anxiety across all ages. They identified 63 potential mediators in six different intervention types but mainly CBT prevention programs were included. Thus, they summarized results on mediators that were mainly connected to the CBT model and clustered them into cognitive, emotional, behavioral, and interpersonal mediator categories with separate lists for children and adolescents. Differences in effective mediators between age groups were found for anxiety but not for depressive disorders. Moreover, they found moderate evidence for mediation effects of cognitive and emotional mediators in adult depression and insufficient evidence for all child/youth mediator studies in depression as well as adult anxiety. Only moderate evidence for cognitive mediators in child/adolescent prevention programs for anxiety were observed. However, only one study fulfilled all requirements for mediation studies and the database was regarded as very limited, because very few studies for each specific mediator variable were available.

Luo and McAloon [30] included 17 RCTs in their meta-analysis and analyzed pooled data from 12 studies on five potential mediators in CBT treatment for childhood anxiety: externalizing difficulties, negative self-talk, coping, fear, and depression. All mediators except fear partially mediated the relation between treatment and outcome in anxiety. However, the study pool was limited for each mediator and the variables did not all address change mechanisms per se as sometimes outcome variables were treated as mediators. The authors concluded that a broader range of potential mediators should be assessed in future research, applying longitudinal designs with multiple points of measurement. Ng et al. [31] concentrated on change mechanisms in the treatment of youth anxiety and depression. They reviewed only studies investigating CBT and Interpersonal Therapy (IPT) and concluded that although four significant candidate mediators could be identified (such as changes in negative cognition, social engagement, family functioning as well as problem solving/ pleasant activities), the evidence was far from being conclusive. They also pointed to the fact that CBT researchers favor cognitive mediators over other possible mediators. Fossum et al. [32] documented long-term treatment effects for children and adolescents with conduct problems and their presumed mediators such as altered cognitions, altered family functioning, or altered parenting in Behavior Therapy, CBT or Family Therapy. Effects of presumed mediators were estimated by calculating the treatment effects and the effect sizes of mediators in a meta-analysis. By comparing effect sizes, Fossum et al. [32] concluded that changes in cognitive mediators had higher effect sizes than changes in family functioning or parenting for adolescents with conduct problems. Again, the proposed mediators did not encompass a range of possible change mechanisms but were limited to the respective therapy model.

In sum, current knowledge about mechanisms of therapeutic change (investigated using statistical mediation) can be described as limited at best for any age group. Almost all studies and reviews are based on CBT intervention studies. Thus, there is a tremendous lack of knowledge regarding evidence-based change mechanisms in other therapeutic approaches. However, even in CBT the evidence base is inconclusive, as many limitations to inferring causality have been observed (e.g., the direction of the effect often remains unclear), mechanisms have not been CBT-specific (e.g., work equally well in the control conditions), and most of the mechanism studies are still unsatisfactory in methodological rigor [28]. Also, the database within the reviews is often strikingly small. Moreover, although the titles of many reviews refer to mechanisms of change or mediator studies, some reviews have only very few real mediation studies included (e.g., three of 33 RCTs in Steubl et al. [27], six in Radu et al. [22], three out of 33 in Fentz et al. [18], 17 out of 30 in Breuninger et al. [17], six of 12 in Stockton et al. [25], or did not include original mediations studies at all [32]). With the exception of the IBI review on depression [26], the original studies were mainly underpowered. The main body of research still focuses on the question of whether the presumed mediator is changed by the intervention—which indeed is the first step in establishing causation, but does not solve the question of therapy or mediator specificity (e.g., Cristea et al. [36]).

The designs and statistical methods used to investigate mechanisms of change are currently very heterogeneous and most studies have not conducted mediation analysis in the control group [17]. For some treatment settings, specific mediators still remain to be articulated or conceptualized (e.g., no specific digital mediators were detected according to Domhardt et al. [26]), and there is a striking lack of studies on the role of therapist behavior on the outcome, or therapist-patient interaction as a mechanism of change [24]. The strongest methodological problems exist with establishing the temporal criterion in mediation analysis (i.e., the mediator has to change before the outcome) as follows: only a minority of studies have accomplished this, and it appears only one original study from 21 reviews directly manipulated a mediator variable in an experimental design so far [37]. Furthermore, studies are missing on younger people, and no review to date has investigated age-specific mediators across diagnoses. Reviews that exclude non-theoretically derived mediators (e.g., Gu et al. [23]) limit the resultant knowledge to mechanisms in line with the therapy model. Yet, due to the complexity of the processes involved, multiple levels of change and change mechanisms are to be expected, rather than just those articulated in the explicit treatment approach [28].

To address these paramount challenges related to mediators and mechanisms of change in effective psychotherapeutic treatment of adolescents, besides other activities, the European Cooperation in Science and Technology (COST) funded a 4-year program named “European Network of Individualized Psychotherapy Treatment of Young People with Mental Disorders” with the acronym TREATme (www.treat-me.eu) that serves as a European multidisciplinary researcher network with researchers and clinicians from 30 countries. A task force within TREATme reviewed the academic research relating to mechanisms of change in patients aged between 10 and 19 years receiving psychological treatments [38]. The current systematic review is the first to summarize the existing knowledge on mediators and theories of change in psychotherapy for adolescents independent of diagnosis or treatment approach and thus the first to follow an age-specific approach that allows drawing firm conclusions for the specific age-group of adolescents. We followed Lemmens et al. [28] to only include studies with a formal mediation test and rated the robustness of evidence and study quality following Moreno-Peral et al. [29]. The aim of this review was to provide an overview of existing research on psychological factors that mediate psychotherapeutic change in adolescents with mental health problems by conducting a narrative synthesis of all studies available to date. The objectives of this review were the following:

1. To identify which mediators and theories of change have been studied in psychotherapy with adolescents
2. To identify if there are adolescence-, disorder- or treatment-specific mediators
3. To critically evaluate the methodological approach of the current research data available on mediators in psychotherapy for adolescents and the robustness of the evidence

## Method

This article is based upon work from the COST Action TREATme (16102). The review was registered in Prospero (CRD42020177535) and follows the preferred reporting items for systematic review and meta-analysis (PRISMA) [39]. The patient/population, intervention, comparison and outcomes (PICO) model [40] was used to define the research question as follows: “In adolescents with psychological problems (P) receiving a psychological intervention (I), what mediators of outcome (O) have been evaluated and found to be significant, when compared to other interventions or control groups (C)“?

### Information sources and search strategy

We included studies from any geographical location, written in English, available as full-text and published from inception until March 23rd, 2022, and which met the specified inclusion criteria (see below). Grey literature such as theses, dissertations or conference proceedings were not included. The search strategy included terms relating to or describing the inclusion criteria. These terms have been combined with the Cochrane MEDLINE filter for controlled trials of interventions and were adapted for PsycINFO [41]. The search string can be found in the online supplementary. The search was performed on the 23rd of March 2022.

### Eligibility criteria and study inclusion

Studies were selected if they included a statistical analysis of mediators in psychotherapy of adolescents within a RCT testing the efficacy of any kind of psychosocial intervention and/or psychotherapeutic intervention. Quasi-experimental, non-controlled, qualitative, cohort, and case studies were excluded. Following the definition of the World Health Organization (WHO), adolescents were regarded as individuals between 10 and 19 years of age, and thus we defined that the majority of study participants must be individuals within this age group. We included studies that reported age means within 10.0–19.9 years, or used age ranges as well as references to school grades that fit the defined age range. In addition, study participants needed to have a mental disorder (e.g., depression, eating disorders) based on DSM or ICD diagnostic criteria, or psychological difficulties (e.g., binge drinking) based on established cut-off values of deployed measures. Studies were included if they reported an intervention aimed at preventing in an at-risk group (i.e., secondary prevention), ameliorating (i.e., tertiary prevention) and/or treating psychological problems of adolescents by using psychosocial mechanisms and strategies in any setting (i.e., individual, family, group, inpatients, E-Mental health, etc.). Examples of interventions included all branches or types of psychotherapy: psychodynamic, integrative, systemic, cognitive-based or cognitive-behavioral, interpersonal, humanistic (such as emotion-focused, supportive, motivational interviewing), psychoeducation and third-wave approaches (such as mindfulness-based therapies). All types of comparators were included (e.g., no intervention, waiting-list, active psychological treatment). Excluded were studies investigating universal or primary prevention programs in which full school cohorts were recruited without specified psychological difficulties or diagnoses. Also, primarily biological or physiological interventions were excluded.

### Evaluating statistical methodology as an inclusion criterion

There is currently no “gold standard” for mediation analysis in psychotherapy research. As such, all studies detailing some form of mediation analysis were considered, and studies were excluded only if no formal mediation test was applied. Nevertheless, the following aspects were taken into consideration when evaluating the included studies and the robustness of the findings.

The extent and significance of the mediation effect depends on the study design outlined above and the type of analytic strategy, which entails different predefined assumptions. Historically, the most commonly used approach in psychology is the causal steps approach based on the work of Baron and Kenny [42]. However, the limitations of this approach include low power and overly low Type I error rates, unless the mediated effect or sample size is large [43]. Yet, often neither of these two conditions is met nor the related assumption that the distribution of the mediated effect is normal [44]. Therefore, several authors have recommended bootstrapping the indirect, mediated effect, which does not involve assumptions about normality and accordingly produces more accurate (and potentially asymmetric) confidence intervals, which yield higher power [45, 46, 47]. A further consideration is the low reliability of measurement instruments, which can be addressed using the structural equation modelling (SEM) framework that allows the estimation of relationships between latent constructs while taking measurement error explicitly into account rather than using imperfect measured indicators confounded with measurement error [48]. Another issue is that in many studies, data may be nested at several levels—such as patients within therapists, therapy groups, or clinics—and if this is ignored, Type I error can be too high. To address this issue, random multilevel modelling of mediated effects has been recommended [49]. It is also possible that the mediated effect varies systematically as a function of another variable (such as age), often termed moderated mediation or mediated moderation [50]. Thus, techniques such as the regions of significance approach have been recommended for identifying ranges of the moderator for which an indirect effect is statistically significant. It should also be noted that while several effect size measures for mediation models have been proposed, these have been considered to require further development [51].

### Study selection process

Study selection was carried out by a group of 20 experienced researchers (doctorate-level) divided into 10 pairs who independently assessed the eligibility of studies retrieved using the search strategy in two phases. The first phase comprised the screening of the titles and/or abstracts of studies that potentially met the inclusion criteria outlined above. In the second phase, each pair of reviewers evaluated the full text of potentially eligible studies to see if they met the inclusion criteria. Disagreements were discussed by the pair. A third reviewer was involved if consensus was not reached. Finally, a fourth independent reviewer (SP) performed an additional quality control check by assessing the eligibility of every fifth excluded study. Disagreements at this stage were solved through discussion with the original review pair.

### Data collection process and data items

Data records were managed using Microsoft Excel. A standardized form was used to collect and extract the information for the review. Extracted information included the following: study setting; study population, participant demographics and baseline characteristics; details of the intervention and control conditions; study methodology; outcomes and times of measurement; assessed mediators; type of mediation analysis; and information for assessment of the risk of bias. Two review authors extracted information independently; discrepancies were identified and resolved through discussion or with a third author when necessary. Another reviewer checked the extracted data for accuracy and finally ST, YY and EH conducted a final check.

### Study quality and risk of bias assessment

Although no standard form for evaluating mediation studies has been established, studies were independently and again in pairs checked for general criteria for identifying mediators of psychosocial interventions in research, such as summarized by Kazdin [13] and Lemmens et al. [28]. Criteria were the following: (1) Is this an RCT? (2) Is there a control group (in which the mediator was also assessed)? (3) Is there a sufficient sample size of *n* ≥ 40 per condition? (4) Are there multiple mediators? (5) Does it meet the temporality criterion (defined by 3 or more assessments of mediator in the treatment phase)? (6) Is there direct experimental manipulation of the mediator?

To further limit risks of bias, a formal risk of bias assessment tool (ROB-tool), the Mixed Methods Appraisal Tool [52], was used to evaluate the overall study quality. This tool permits the appraisal of the methodological quality of five categories of studies of which we used only the quality rating for RCTs with 6 items covering the appropriateness of research questions and the research design, quality of randomization, blinding of assessors as well as adherence to treatment.

All items from the mediator study quality as well as the RCT risk of bias assessment were coded with either “Yes”, “No” or “Unclear”. A “Yes” rating was given one point, while “No” and “Unclear” were given no points. This sums up to a maximum of six points for mediator study quality and also a maximum of 6 points for the overall study quality with the ROB-tool. In a second step, all studies were classified for study quality into three categories: good (risk of bias 5 to 6 AND mediator study quality 5 to 6), satisfactory (risk of bias 5 to 6 AND mediator study quality 4) and unsatisfactory (risk of bias lower than 5 OR mediator study quality lower than 4).

### Synthesis methods

To summarize the current state of mediator research, we conducted a narrative synthesis of all mediator constructs used from the included studies. During the data extraction process, the construct as well as the measure of mediator was collected. Using qualitative content analysis [53], all mediator constructs were first categorized into broad categories and second, assigned to a sub-category. This process was done iteratively, by revisiting the material twice when categorization was completed and discussion between authors ST and YI.

In order to draw narrative conclusions from our findings, we used the adaptation of the Best Evidence Synthesis Rating System (BESRS) that was presented by Moreno-Peral et al. [29]. Therefore, the number of studies that evaluated the same mediator-construct for the same disorder category (in at least three studies), the statistically significant association criteria for mediation, and the methodological quality of each of the studies (good/ satisfactory/ unsatisfactory) were taken into account. The scientific evidence was categorized into three levels as follows: (a) *strong evidence* (at least 65% of the potential mediators are significantly associated with change across at least three RCTs with the quality being between good and satisfactory); (b) *moderate evidence* (at least 65% of the potential mediators are statistically significant across at least three RCTs with the quality being mixed between good, satisfactory and unsatisfactory); and (c) *insufficient evidence* (< 65% of the potential mediators are statistically significant or at least three independent studies have not been identified, or at least 65% of the potential mediators are statistically significant across at least three RCTs but all of them have unsatisfactory quality rating). Therefore, levels of scientific evidence were based on counting the number of significant results and weighting the quality of the studies.

### Ethics

No ethical approval was necessary to conduct the review as no additional empirical data were assessed.

## Results

**Fig. 1:**
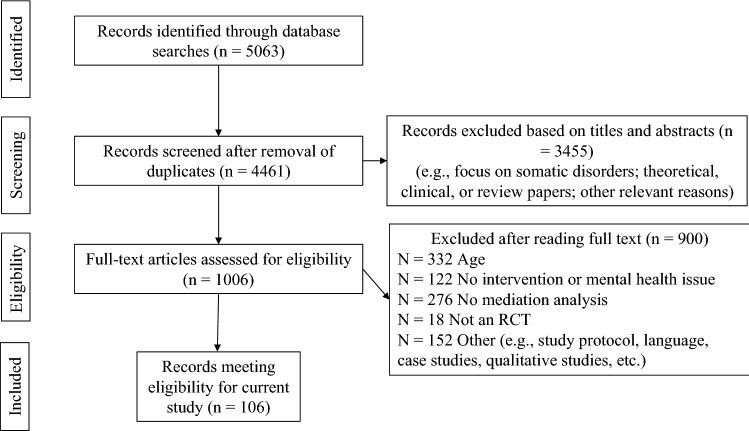
PRISMA Flowchart

The search in MEDLINE and PSYCHINFO identified 5063 papers of which 4461 remained after removing duplicates (cp. Fig. 1). After screening of abstract and titles, 1006 publications were reviewed in full text. Exclusion criteria after reading full texts were mainly due to not fulfilling the age range criteria, not including a psychosocial intervention of any kind, not performing formal mediation tests and not including a RCT. Finally, 106 studies fulfilled the eligibility criteria of the present review and data from these were extracted.

Table 2 summarizes all 106 studies included with regard to main author, year of publication, country where the study was conducted, diagnoses or psychological problem, characteristics of the participants, treatment and comparator, mediation statistics and mediator categories as well as mediator significance. The summary table is grouped by diagnosis. The specific diagnoses were categorized in broader types if more than five studies addressed a specific disorder range. This yielded the followingsix diagnostic categories: anxiety (*k* = 19), depression (*k* = 20), externalizing disorders including offending and criminal behavior (*k* = 14), substance use disorders including alcohol, cannabis, cigarettes and other drug use (*k* = 25), posttraumatic stress disorders (*k* = 8) and other diagnoses (*k* = 22) for diagnoses that were only studied in one to three RCTs (e.g. eating disorders, OCD, ADHD, irritable bowel syndrome, etc.). Two studies were dedicated to both depression and anxiety equally [54, 55] and were thus counted for both diagnoses.

**Table 2:** Summary of studies included in the review and study characteristics

Authors	Year	Country	Diagnosis	Mean age (SD)	% Female	Name of intervention	Type of intervention	Setting	Delivery mode	Length in weeks	Session number	Comparator	Sample size (N)	Mediator category and significance
Alfano et al.	2009	USA	Anxiety	11.20 (2.35)	48.9	Social Effectiveness Therapy for Children (SET-C)	CBT	IND; GRO	F-2-F	12	12	Education / Pharmacotherapy / Placebo	88	EMO↑ REL↓
Allan et al.	2015	USA	Anxiety	18.8 (1.5)	82.9	Anxiety Sensitivity Education and Reduction Training (ASERT) program; control: Physical Health/Education Training (PHET)	CBT; EDU	IND	BLENDED	1	1	Education	82	COG↑ COG↓ COG↓ COG↑
Blake et al.	2017	Australia	Anxiety	14.48 (0.95)	60	Sleep SENSE: cognitive behavioral and mindfulness-based sleep intervention	CBT; MIN	GRO	F-2-F	7	7	Active psychological intervention	123	BEH↑ COG↑ BEH↓
Blake et al.	2017	Australia	Anxiety	14.48 (0.95)	60	Sleep SENSE: cognitive behavioral and mindfulness-based sleep intervention	CBT; MIN	GRO	F-2-F	7	7	Active psychological intervention	123	BEH↓ BEH↓ BEH↑
Chu et al.	2004	USA	Anxiety	8 to 14	41.3	Cognitive–behavioral treatment for children with anxiety (CBT)	CBT	IND	F-2-F	16	16	NR	59	BEH ↓ BEH↑
Fjermestad et al.	2020	Norway	Anxiety	11.5	52.1	FRIENDS for Life	CBT	IND	F-2-F	10	10	NR	73	REL↑
Hogendoorn et al.	2014	Nether-lands	Anxiety	12.51 (2.83)	57	Cognitive behavioral therapy with the Coping Cat protocol	CBT	IND; FAMI	F-2-F	12	12	Waiting list	113	COG↑ BEH↑ COG↑
Kwok	2019	Hong Kong	Anxiety	13.5	35.8	Positive Psychology and Music Therapy	OTH	GRO	F-2-F	8	8	No intervention	106	COG↑ EMO↓
Norr et al.	2014	USA	Anxiety	18.9 (1.42)	83.7	Anxiety Sensitivity Education and Reduction Training (ASERT)	EDU; CBT	IND	ONLINE	1	1	Education	104	COG ↑EMO↑ EMO↓
Norr et al.	2017	USA	Anxiety	19.09	85.5	Cognitive Anxiety Sensitivity Treatment (CAST)	CBT	IND	ONLINE	1	1	Education	54	COG↑ COG↓ COG↓
Ollendick et al.	2017	USA & Sweden	Anxiety	10.29	38	One-Session-Treatment (OST)	CBT	IND	F-2-F	1	1	Education	165	COG↑ COG↑ BEH↑
Schleider et al.	2015	USA	Anxiety	10.7	50	Cognitive behavioral treatment (Coping Cat program)	CBT	IND; FAMI	F-2-F	12	14	Pharmacotherapy / Active psychological intervention and pharmacotherapy / Placebo	433	FAM↑ FAM↑
Smits et al.	2008	USA	Anxiety	19.9	18–51	Exercise (Ex) and exercise plus cognitive restructuring (Ex + C)	OTH	IND	F-2-F	2	6	Waiting list / Active psychological intervention	60	COG↑
Swain et al.	2015	Australia	Anxiety	13.8 (1.4)	63.3	Acceptance and Commitment Therapy (ACT)	MIN	GRO	F-2-F	10	10	Active psychological intervention / Waiting list	49	COG↑ COG↓ COG↓
Timpano et al.	2016	USA	Anxiety	18.9	83.7	Anxiety sensitivity education and reduction training (ASERT)	CBT	IND	F-2-F	1	1	Education	104	COG↑
Wu et al.	2020	USA	Anxiety	10.8	51.6	Cognitive-behavioral therapy plus the Coping cat (CBT)	CBT	IND	F-2-F	14	14	Active psychological intervention and pharmacotherapy	279	BEH↑
Fjermestad et al.	2016	Norway	Anxiety	11.4 (2.1)	50	Cognitive-behavioral therapy (CBT)	CBT	IND	F-2-F	14	12	Waiting list	91	REL↓ REL↓ REL↑
Topper et al.	2017	Netherlands	Anxiety, Depression	17.32 (1.97)	83	Rumination-focused cognitive-behavioral-therapy (RFCBT)	CBT	GRO	BLENDED	6	6	Treatment as usual	241	COG↑
Yap et al.	2019	Australia	Anxiety, Depression	13.68 (1,06)	45	Partners in Parenting	INT	IND	ONLINE	12	9	Education	317	FAM↑
Andrew et al.	2015	USA	Depression	14.8 (1.6)	41.5	Behavior Family Systems Therapy (BFST)	INT	FAMI	BLENDED	12	12	NR	82	FAM↑
Brent et al.	1998	USA	Depression	13 to 18	NR	Cognitive-behavioral-therapy (CBT), systemic-behavioral family therapy (SBFT), nondirective supportive therapy (NST)	CBT	IND	F-2-F	12 to 16	12 to 16	Active psychological intervention	78	COG ↑
Brunwasser et al.	2018	USA	Depression	12.5 (11.9)	46.1	Penn Resiliency Program (PRP)	CBT	GRO	F-2-F	12	12	Education / Placebo / No intervention	321	COG↑ THERA↑
Compas et al.	2010	USA	Depression	11.4 (1.9); 11.3 (2.1)	42	Family group cognitive-behavioral (FGCB) preventive intervention	CBT	FAMI; GRO	F-2-F	24	12	Guided self-help	111	BEH↑ FAM↑ FAM↓
Dietz et al.	2014	USA	Depression	15.6 (1.3)	77.8	Cognitive behavior therapy (CBT), systemic behavior family therapy (SBFT), nondirective-supportive therapy (NST)	CBT; SYS	IND	F-2-F	12 to 16	12 to 16	Active psychological intervention	63	REL↑ COG↑
Fosco et al.	2016	USA	Depression	11 to 16	49	Family Check-Up (FCU)	HUM	FAMI	F-2-F	NR	3	No intervention	386	FAM↑
Gladstone et al.	2014	USA	Depression	17.5 (2.04)	56.2	Competent Adulthood Transition with Cognitive-behavioral, Humanistic and Interpersonal Training (CATCH-IT)	INT	IND	ONLINE	NR	14	Education	69	COG↑ REL↓ FAM↓
Jacobs et al.	2009	USA	Depression	14.36 (1.5)	54	Cognitive-behavioral therapy (CBT)	CBT	IND	F-2-F	12	12	Pharmacotherapy / Active psychological intervention and pharmacotherapy / Placebo	439	COG↑ COG↓
Jones et al.	2019	USA	Depression	14.01	66.7	Interpersonal Psychotherapy–Adolescent Skills Training (IPT-AST)	IPT	GRO; IND; FAMI	F-2-F	NR	15	Active psychological intervention	183	FAM↑ REL↓ REL↓ REL↑ REL↑
Kauer et al.	2012	Australia	Depression	18.5 (3.2)	87.9	Mobile Tracking of Young People's Experiences	EDU	IND	ONLINE	2	28	Guided self-help	114	EMO↑
Kaufman et al.	2005	USA	Depression	15.1 (1.4)	48.4	Adolescent Coping With Depression (CWD-A)	CBT	GRO	F-2-F	8	16	Education	93	COG↑
Lewis et al.	2009	USA	Depression	14.6 (1.5)	54.4	Cognitive-behavioral therapy (CBT)	CBT	IND	F-2-F	12	15	Pharmacotherapy / Active psychological intervention and pharmacotherapy / Placebo	253	BEH↑
Mehlum et al.	2014	Norway	Depression	15.6 (1.5)	88.3	Dialectical Behavior therapy for adolescents (DBT-A)	MIN	IND; FAMI	F-2-F	19	48	Treatment as usual	77	THERA↓ THERA↑
Rossouw et al.	2012	UK	Depression	14.7 (1.3)	85	Mentalization-Based-Treatment for Adolescents (MBT-A)	PTD	IND; FAMI	F-2-F	52	52	Treatment as usual	80	COG↓ REL↑
Smith et al.	2015	UK	Depression	12 to 16	NR	Computerised-Cognitive-behavioral therapy (C-CBT)	CBT	IND	ONLINE	8	8	Waiting list	110	COG↑
Stice et al.	2011	USA	Depression	15.5 (1.2)	57	Group cognitive behavioral depression prevention intervention (CB)	CBT	GRO	F-2-F	6	6	Guided self-help / Education	253	THERA↑
Stice et al.	2010	USA	Depression	15.6 (1.2)	NR	(1) cognitive behavioral group (CB); (2) cognitive behavioral bibliotherapy; (3) supportive expressive group	CBT; CBT; HUM	GRO	F-2-F	6	NR	No intervention	341	COG↑ BEH↑ EMO↑ EMO↑
Zhou et al.	2020	USA	Depression	14.8 (1.8)	77.5	Interpersonal Therapy for Adolescents (IPT-A)	IPT	IND	F-2-F	16	12–16	Active Psychological Intervention and Pharmaco-Treatment	40	REL↑
Borduin et al.	2021	USA	Externalizing Disorder	14.0 (1.9)	NR	Multisystemic Therapy for problem sexual behaviors (MST-PSB)	SYS	FAM	F-2-F	31	90	Treatment as usual	48	REL↑ REL↑ FAM↑
Dadds et al.	2012	Australia	Externalizing Disorder	10.5	25	Emotion recognition training (ERT)	SYS	IND; FAMI	F-2-F	4	4	Treatment as usual	195	EMO↓ EMO↓
Dekovic et al.	2012	Netherlands	Externalizing Disorder	16.02 (1.31)	26.56	Multisystemic therapy (MST)	SYS	FAMI	F-2-F	16 to 24	NR	Treatment as usual	256	FAM↑ FAM↑ FAM↓ FAM↓
Eddy et al.	2000	USA	Externalizing Disorder	14.9(1.3)	0	Multi-Dimensional Treatment Foster Care (MTFC) and group care (GC)	SYS	FAMI; IND; GRO	F-2-F	NR	NR	Treatment as usual	79	FAM↑ FAM↑ FAM↑ REL↑
Henggeler et al.	2009	USA	Externalizing Disorder	14.6 (1.7)	2.4	Multisystemic therapy (MST)	SYS	FAMI	F-2-F	28	28	Treatment as usual	121	FAM↑ REL↑ THERA↓ FAM↓ REL↓
Henggeler et al.	1992	USA	Externalizing Disorder	15.2 (1.4)	23	Multisystemic therapy (MST)	SYS	FAMI	F-2-F	13	NR	Treatment as usual	84	FAM↓ REL↓ THERA↓ FAM↓ REL↓
Hogue et al.	2006	USA	Externalizing Disorder	15.47 (1.31)	19	Cognitive–behavioral therapy (CBT) or multidimensional family-based therapy (MDFT)	CBT; SYS	IND; FAMI	F-2-F	16 to 24	16 to 24	Active psychological intervention	100	REL↑ FAM↑
Huey et al.	2000	USA	Externalizing Disorder	14.6 (1.5); 15 (1.1)	Sample 1: 17 and sample 2: 20	Multisystemic therapy (MST)	SYS	FAMI	F-2-F	NR	NR	Treatment as usual	115	THERA↑ FAM↑ FAM↑ FAM↑ REL↑
Lindsey et al.	2019	USA	Externalizing Disorder	9 to 12	33	Coping Power intervention	CBT	GRO	F-2-F	NR	34	NR	118	BEH↑ FAM↑ BEH↑
Pantin et al.	2009	USA	Externalizing Disorder	13.8 (0.76)	36.15	Familias Unidas	EDU	FAMI	F-2-F	NR	19	No intervention	213	FAM↓ FAM↑ FAM↓ FAM↑ FAM↑
Paquette et al.	2014	Canada	Externalizing Disorder	19.99(2.41); 19.54 (2.32)	12.27	Wilderness Therapy program Chance for Change	EDU	GRO	F-2-F	2	NR	Active psychological intervention	220	REL↓ BEH↓
Perrino et al.	2016	USA	Externalizing Disorder	14.7 (1.38)	35.54	Familias Unidas	EDU	FAMI	F-2-F	12	7	No intervention	232	FAM↑
Van Ryzin et al.	2012	USA	Externalizing Disorder	15.31 (1.17)	100	Multidimensional treatment foster care (MTFC)	SYS	FAMI; IND	F-2-F	25	NR	No intervention	153	REL↑
Werch et al.	2011	USA	Externalizing Disorder	grade 11 and 12	61.6	Project Active (Brief Integrated multiple behavior intervention)	EDU	IND	BLENDED	4	NR	Treatment as usual	451	COG↑ REL↑ COG↑ COG↑
Asarnow et al.	2021	USA	Other (Self-Harm)	14.89 (1.47)	12–18	Dialectic Behavioral Therapy (DBT)	MIN	IND, FAM	F-2-F	26	52	Active Psychological Intervention	95	EMO↑, FAM↓, BEH↓
Boyer et al.	2018	Netherlands	Other (ADHD)	14.48 (1.21)	27.5	Plan my life (PML: planning-focused) and Solution-focused treatment (SFT)	CBT; HUM	IND	F-2-F	10	10	Active psychological intervention	69	REL↑ REL↓
Forsberg et al.	2017	USA	Other (Anorexia Nervosa)	14.4 (1.6)	91	Family-based treatment (FBT) for anorexia nervosa and adolescents-focused therapy (AFT) for anorexia nervosa	SYS	FAMI; IND	F-2-F	54	24	Active psychological intervention	224	FAM↑ FAM↓
Le Grange et al.	2012	USA	Other (Anorexia Nervosa)	14.4 (1.6)	91	Individual adolescent focused therapy (AFT) and family based therapy (FBT)	SYS	IND	F-2-F	32	32	Active psychological intervention	100	COG↓ THERA↓ COG↓ BEH↓ FAM↓
Tein et al.	2006	USA	Other (bereaved children)	11.39	100	Family bereavement program (FBP)	CBT	FAMI; IND	F-2-F	12	12	Guided self-help	156	FAM↑ BEH↑ COG↑ EMO↑ COG↑
Goldstein et al.	2020	USA	Other (Bipolar disorder)	16.6 (2.4)	53,5	Brief motivational intervention (BMI)	HUM	IND	F-2-F	4	3	Treatment as usual	40	THERA↑
Summers et al.	2016	USA	Other (Body Dysmorphic Disorder)	19.63; 19.95	IG 84.2, CG 73.7	Interpretation bias modification training (IBM)	CBT	IND	ONLINE	2	4	Placebo	38	COG↑
Stice et al.	2007	USA	Other (Body image concerns)	17.1 (1.4)	100	Dissonance intervention, Healthy weight intervention	CBT, EDU	GRO	F-2-F	3	3	Guided self-help	340	COG↑ BEH↓ BEH↓
Harrington et al.	2000	UK	Other (Deliberate self-poisoning)	10 to 16	NR	Brief family-based intervention	SYS	FAMI; IND	F-2-F	4	5	Treatment as usual	162	FAM↓ COG↓ FAM↓ COG↓ THERA↓
Tan et al.	2015	Australia	Other (divers)	15.40 (1.55)	75	Taming the Adolescent Mind (TAM)	MIN	GRO	F-2-F	5	5	Treatment as usual	80	COG↑
Seidel et al.	2009	USA	Other (High risk for eating disorders)	19.8 (1.3)	100	Dissonance intervention	CBT	GRO	F-2-F	4	4	No intervention	71	COG↑ COG↑
Bruin et al.	2018	Netherlands	Other (Insomnia)	15.6 (1.6)	75	Cognitive behavioral therapy for insomnia (CBTI): Internet (CBTI-IT) and face to face group treatment (CBTI-GT)	CBT	IND; GRO	BLENDED	6	7	Waiting list/Active psychological intervention	116	BEH↑
Bonnert et al.	2018	Sweden	Other (Irritable bowel syndrome)	15.54 (1.56)	61	Exposure-based internet delivered cognitive-behavioral therapy (Internet CBT)	CBT	IND; FAMI	ONLINE	10	10	Waiting list	101	BEH↑ EMO↓
Kashikar-Zuck et al.	2013	USA	Other (Juvenile fibromyalgia)	15.02 (1.75)	NR	Cognitive behavioral therapy (CBT) and fibromyalgia education (FE)	CBT	IND; FAMI	F-2-F	8	10	Education	100	BEH↓ COG↓ BEH↓
Orkibi et al.	2017	Israel	Other (loneliness)	14.5 (0.78)	40	Psychodrama group therapy (PD)	HUM	GRO	F-2-F	16 to 22	16 to 22	Waiting list	13	BEH↑ EMO↑ COG↑ THERA↓ BEH↑
Bakhshaie et al.	2020	USA	Other (OCD)	12.39 (2.92)	51,4	Exposure and response prevention (ERP)	CBT	IND	F-2-F	8	10	Active psychological intervention and pharmacotherapy	139	THERA↑ THERA↓
Peris et al.	2017	USA	Other (OCD)	12.71	43	Positive Family Interaction Therapy (PFIT)	CBT	IND; FAMI	F-2-F	12	18	Treatment as usual	62	FAM↑
Weintraub et al.	2021	USA	Other (Bipolar)	13.2 (2.6)	65.5	Family-focused therapy (FFT)	SYS	FAM	F-2-F	16	12	Education	119	FAM ↑ REL↑
Wolters et al.	2019	Netherlands	Other (OCD)	12.8	58.6	Control your OCD	CBT	IND	F-2-F	16	16	No intervention	58	COG ↓
Mehlum et al.	2019	Norway	Other (Suicidal and self‐harming behavior)	18.79 (1.61)	90	Dialectical Behavior Therapy (DBT-A)	CBT	IND	F-2-F	19	NR	NR	77	COG↑
Pineda et al.	2013	Australia	Other (Suicidal behavior)	15.4 (1.23)	75.5	Resourceful Adolescent Parent Program (RAP-P)	EDU	FAMI	F-2-F	NR	4	No intervention	48	FAM↑ FAM↑
Czyz et al.	2019	USA	Other (Suicide risk)	15.42 (1.36)	78,8	Motivational interviewing enhanced safety planning (MI-SafeCope)	HUM	IND, FAMI	F-2-F	8	2	Treatment as usual	34	BEH↑ BEH↑ COG↑ BEH↓ FAM↓
Meiser‐Stedman et al.	2017	UK	Post-Traumatic-Stress-Disorder	13.3 (2.5)	72.4	Cognitive Therapy for PTSD (CT for PTSD)	CBT	IND	F-2-F	10	8	Waiting list	26	COG↑ COG↓ BEH↓ BEH↑
Kangaslampi et al.	2016	Palestina	Post-Traumatic-Stress-Disorder	11.29 (0.68)	50	Teaching recovery techniques intervention (TRT)	CBT	GRO	F-2-F	4	8	Waiting list	433	COG ↓
Jensen et al.	2018	Norway	Post-Traumatic-Stress-Disorder	15.1	79.5	Trauma-focused cognitive-behavioral therapy (TF-CBT)	CBT	IND, FAMI	F-2-F	NR	13	Treatment as usual	153	COG↑
Pfeiffer et al.	2017	Germany	Post-Traumatic-Stress-Disorder	12.80—13.23	73	Trauma-Focused CBT (TF-CBT)	CBT	IND; FAMI	F-2-F	12	12	Waiting list	123	COG ↑
Smith et al.	2007	UK	Post-Traumatic-Stress-Disorder	13.89	50	Cognitive-behavioral-therapy (CBT)	CBT	IND; FAMI	F-2-F	10	10	Waiting list	24	COG ↑
McLean et al.	2015	USA	Post-Traumatic-Stress-Disorder	15.3 (1.5)	100	Prolonged exposure therapy for adolescents (PE-A)	CBT	IND	F-2-F	8 to 14	8	Active psychological intervention	53	COG↑
Knutsen et al.	2018	Norway	Post-Traumatic-Stress-Disorder	15.0 (2.2)	74.7	Trauma-focused cognitive-behavioral therapy (TF-CBT)	INT; SYS; CBT	IND; FAMI	F-2-F	12	12 to 15	Treatment as usual	36	COG↑ THERA↓
Tutus et al.	2019	Germany	Post-Traumatic-Stress-Disorder	13.1 (2,82)	73.5	Trauma-focused cognitive-behavioral therapy (TF-CBT)	CBT	IND	F-2-F	12	12	No intervention	113	FAM↑
Black et al.	2012	USA	Substance Use Disorder	19.0	39	Brief Intervention for Socially Anxious Drinkers (BISAD)	CBT	GRO	F-2-F	3	3	Education	41	COG↑ BEH↑
Gonzalez et al.	2012	USA	Substance Use Disorder	12.3 (0.54)	50.8	Bridges/puentes	OTH	GRO; FAMI	F-2-F	9	11	Education	516	BEH↑BEH↑ FAM↑ FAM↑
Barnett et al.	2014	USA	Substance Use Disorder	16.7	30	Motivational interviewing (MI)	HUM	GRO; IND	BLENDED	36 to 48	3	Active psychological intervention	122	BEH↑
Barnett et al.	2007	USA	Substance Use Disorder	18.8 (0.87)	51.1	Brief Motivational Interview (BMI) or computer-delivered intervention (CDI)	HUM	IND	F-2-F	1	1	Active psychological intervention	212	BEH↑
Borsari et al.	2000	USA	Substance Use Disorder	18.45 (0.11)	59	Brief Motivation lntervention (BMI)	HUM	IND	F-2-F	1	1	No intervention	60	COG↑
Borsari et al.	2015	USA	Substance Use Disorder	18.83 (0.81)	NR	Brief Motivation lntervention (BMI)	HUM	IND	F-2-F	1	1	Education	249	THERA↑ COG↑ REL↓ THERA↑
Botvin et al.	1995	USA	Substance Use Disorder	14.96	53	Generic skills training (GSI) and culturally focused intervention (CFI)	CBT; EDU	GRO	F-2-F	8	15	Active psychological intervention/Education	456	COG↑ BEH↑ BEH↑
Brody et al.	2012	USA	Substance Use Disorder	17.7	58.5	Adults in the Making (AIM)	CBT; EDU	GRO	F-2-F	6	4	NR	289	COG↑ BEH↑
Carey et al.	2018	USA	Substance Use Disorder	19.2 (1.16)	28	Brief motivational interviewing (BMI)	HUM	IND	BLENDED	1	1	Active psychological intervention/Education	554	COG↓ REL↓ COG↓
Chaplin et al.	2021	USA	Substance Use	13.89 (1.69)	51	Parenting Mindfully [PM] intervention	MIN	GRO	F-2-F	8	8	Education	96	FAM↑ FAM↑
Chen et al.	2017	USA	Substance Use Disorder	11 to 16	NR	Strong African American families program (SAAF)	INT	FAMI	F-2-F	NR	7	Education	424	FAM↑
D'Amico et al.	2015	USA	Substance Use Disorder	16.75	34.5	Free Talk (FT) motivational interviewing group	HUM	GRO	F-2-F	6	5.3	Treatment as usual	110	THERA↑ THERA↑ COG↓
Diamond, et al.	2006	USA	Substance Use Disorder	15.7 (1.2)	19	Motivational Enhancement Therapy (MET), Cognitive-behavioral group therapy (CBT5), Family Support Network (FSN), Adolescent Community Reinforcement Approach (ACRA), Multidimensional Family therapy (MDFT)	CBT; HUM; SYS	IND; FAMI	F-2-F	5; 12	5; 12	Active psychological intervention	356	RE↑ REL↓
Doumas et al.	2009	USA	Substance Use Disorder	19.24 (1.33)	27.6	Web-based personalized normative feedback (WPNF)	HUM; EDU	IND	ONLINE	1	1	Education	67	COG↑
Dunn et al.	2019	USA	Substance Use Disorder	19.42	33	Brief motivational interviewing (BMI) with personalized normative feedback (PNF) or Expectancy Challenge Alcohol Literacy Curriculum (ECALC)	HUM	IND	ONLINE	1	1	Active psychological intervention	121	COG↑
Kenney et al.	2014	USA	Substance Use Disorder	18.07 (0.54)	100	Protective behavioral strategies (PBS) skill training	CBT	GRO	F-2-F	1	1	Education	226	THERA↑
Magill et al.	2017	USA	Substance Use Disorder	18.2 (0.98)	58	Motivational Interview (MI)	HUM	IND	F-2-F	1	1	Education	167	BEH↑ COG↑ COG↑ COG↑ COG↑
Magill et al.	2019	USA	Substance Use Disorder	18.2 (0.98)	62	Brief Motivational Intervention (BMI)	HUM	IND	F-2-F	1	1	Active psychological Intervention	165	BEH↑ THERA↓ THERA↓
McNally et al.	2005	USA	Substance Use Disorder	18.58 (0.78)	71	Motivationally based intervention (MBI)	HUM	IND	F-2-F	1	1	No intervention	73	COG↓ COG↓
Murphy et al.	2012	USA	Substance Use Disorder	18.5 (0.71)	50	Brief motivational interviewing (BMI)	HUM	IND	F-2-F	1	1	Active psychological intervention/Education	82	BEH↑ COG↓
Murphy et al.	2019	USA	Substance Use Disorder	18.77 (1.06)	61	Brief motivational interviewing (BMI) + Substance-fee Activity Session (SFAS), Relaxation training (RT)	HUM	IND	BLENDED	2	2	No intervention	393	BEH↑ BEH↓ THERA↓
O'Leary-Barrett et al.	2017	Canada	Substance Use Disorder	12 to 13	NR	Personality-targeted interventions	CBT; EDU; HUM	GRO	F-2-F	2	2	No intervention	154	COG↑ BEH↑ COG↑
Orlando et al.	2005	USA	Substance Use Disorder	grade 7 and 8	50	Project ALERT	CBT	GRO	F-2-F	14	14	No intervention	4277	REL↑ COG↑
Winters et al.	2012	USA	Substance Use Disorder	16.3	48	Brief Intervention adolescent only (BI-A) or brief intervention adolescent and additional parent session (BI-AP)	HUM	IND; FAMI	F-2-F	2	2	Active psychological intervention/No intervention	315	BEH↓ COG↑ FAM↓ THERA↑
Winters et al.	2014	USA	Substance Use Disorder	16.06 (1.4)	50.4	Brief Intervention adolescent only (BI-A) or brief intervention adolescent and additional parent session (BI-AP)	HUM	IND; FAMI	F-2-F	2	2	Active psychological intervention/No intervention	284	BEH↑ COG↓ FAM↑ THERA↑

Legend: ↑: significant mediation effect, ↓: non-significant mediation effect

*BEH* behavioral mediator, *BLENDED* blended treatment with face-to-face and online elements, *COG* cognitive mediator, *CBT* cognitive-behavioral therapy, *EDU* educational intervention, *EMO* emotional mediator, *FAM* family-related mediator, *FAMI* family treatment, *F-2-F* face-to-face treatment, *GRO* group treatment, *HUM* humanistic therapy, *IND* individual treatment, *INT* integrative therapy, *IPT* interpersonal therapy, *ONLINE* online treatment, *MIN* mindfulness-based therapy, *OTH* other therapy approaches, *PDT* psychodynamic therapy, *REL* relational mediator, *SYS* systemic therapy, *THERA* therapy-related mediator

The mean age of participants ranged from 10.29 to 19.99 years. When mean age was not reported, the age range was used instead. In some cases, authors reported only school grades. We decided to include studies with school grades within the age range of 10 and 19, starting with grades five to six. Studies varied between 0 and 100% of female participants. Across all studies, a ratio of 57% of participants was female. Sample sizes ranged between 13 and 4277 participants with a mean of 170 participants (when the outlier of 4277 participants was excluded). In total, data for 19,407 participants were included in this review.

The included studies were from 11 different countries. A huge majority of studies were conducted in the USA (*k* = 73, 68%), followed by *k* = 8 from Australia (7.5%) and *k* = 6 (5.7%) each from the Netherlands and Norway and five from the UK; there were also two studies each from Canada, Germany and Sweden (one Swedish study was co-joint with the USA). One study each was included from Israel, Palestine and Hong Kong.

The interventions included in the studies were diverse and categorized into eight different approaches as folows: Cognitive-Behavioral Therapy (CBT with *k* = 54, 51%), Humanistic Therapies (HUM with *k* = 23, 22%), Systemic Therapies (SYS with *k* = 16, 15%), Educational Approaches (EDU with *k* = 14, 13%), Interpersonal Therapy (IPT with *k* = 2, 1.8%), third wave mindfulness therapies (MIN with *k* = 5, 5%) and other approaches (OTH with *k* = 3, e.g. Positive Psychology or culturally adapted programs). Five studies investigated an explicitly integrative treatment approach and one study included psychodynamic therapy (PDT). Settings differed in terms of individual, group or family therapy or a combination, respectively: 68 studies reported on treatments that included individual therapy for adolescents of which *k* = 41 offered only individual therapy, *k* = 22 offered individual therapy in conjunction with family sessions, and *k* = 5 offered individual therapy in conjunction with group sessions and/or family treatment. Group treatment was investigated in *k* = 31 studies in which *k* = 24 had group treatment only, and in the other studies, group treatment was combined with either individual and/or family treatment. Family therapy without additional settings was only conducted in *k* = 12 studies. All studies investigated outpatient treatment using a face-to-face (*k* = 88), online (*k* = 10) or blended (*k* = 8) mode of delivery. Treatment length in weeks varied from one to 54 weeks with a mean duration of 8.7 weeks. Session amount ranged from one to 52 sessions with a mean session count of 8.6 sessions across all studies. 24 studies reported on treatments equal to or shorter than four weeks and 12 studies reported on single-session interventions. Studies also differed in their choice of comparator conditions, which included no intervention (*k* = 14, 13%), waiting list (*k* = 13, 12%), treatment as usual (*k*  = 19, 18%), placebo (*k*  = 3, 3%), pharmacotherapy (*k* = 7, 6.6%) education (*k*= 24, 22.6%), guided self-help (*k* = 5, 4.7%) and active psychological treatments (*k*  = 31, 29%).

In terms of general statistical models for mediation tests, the following methods were extracted from the included studies: regression models (*k* = 43, 40.6%), structural equation modeling (SEM) (*k* = 20, 19.6%), path models (*k* = 13, 12.7%), hierarchical linear modeling (HLM; *k* = 14, 13.2%), general linear mixed model (GLM; *k* = 6, 5.8%), growth curve analysis (GCA; *k* = 4, 3.9%), multilevel regression analysis (MRA; *k* = 4, 3.9%) as well as cross lagged panel analysis (CLP; *k* = 2, 1.9%). Different estimation techniques and/or programs were reported to calculate the indirect effect of the mediator variable on outcome such as bootstrapping (*k* = 42, 39.6%), Baron & Kenny (B & K; *k* = 10, 9.8%), Sobel test or delta method (*k* = 7, 6.8%), PRODCLIN (*k* = 7, 6.8%), joint significance (*k* = 3, 2.9%), singular use of asymmetric distribution of products, marginal mediation models, and rank preserving models. However, *k* = 34 (32.1%) studies did not clearly report a specific estimation technique.

### Qualitative synthesis of mediators

We identified a total of 252 mediators that were analyzed in the 106 included studies. With the exception of seven mediation analysis, all mediators were investigated in relation to the respective primary outcome. Of the exceptions, five were investigated only as mediators of a secondary outcome and two were investigated as mediating a process variable such as change talk. In 168 analyses, the mediation effect was reported to be significant whereas in 84 analyses the mediation effect was reported to be non-significant. The majority of studies assessed multiple mediators (*k* = 69, 65%); however, only 8 studies performed a multiple mediator testing whereas the other studies performed mediation analysis individually for each mediator. A narrative synthesis of all mediators using qualitative content analysis led to six different categories of mediators (number of mediation tests that were reported for the respective mediator): cognitive (80), family-related (54), behavioral (48), therapy-related (34), relational (23), and emotional (13) (compare Table 3). No biological mediator was identified in adolescent therapy changes in the current literature. Comparable to other reviews, cognitive mediators were most commonly investigated. However, in contrast to mediator research in adults, behavioral, relational and family-related mediators were also investigated in a large number of studies. Only very few studies investigated emotional mediators. With regards to the measures used to assess putative change mechanisms, we identified 181 different measures that were used in the studies. Identical mediator measures were very seldom employed across different studies; exceptions with at least three different mediation analyses using the same measure were the following: Anxiety Sensitivity Index-3 (ASI-3, [56], Children’s Post-Traumatic Cognitions Inventory (CPTCI; [57]). Symptom-Check-List (SCL-90, [58]), and Therapeutic Alliance Scale for Children (TASC-C/T, [59]). Furthermore, virtually all measures were self-reports except for some observer-rated measures mainly from therapy studies about Motivational Interviewing (e.g., Motivation to Change, Therapist Technique and Involvement in Therapy). The constructs within each mediator type were further subcategorized using qualitative content analysis. The relation between significant and non-significant results is reported for each category and sub-category to identify promising mediators defined as having more than 65% statistically significant mediation effects.

**Table 3:** Mediator qualitative synthesis

Mediator (*n*)	Constructs	Number (m) and type of measures	Significance	
			Yes (*n*)	No (*n*)
Total (252)			160	82
Cognitive (80)			57	23
Anxiety sensitivity (11)	Anxiety sensitivity, cognitive, physical and social concerns, perceived control over anxious situations	*m* = 2 (self-report), mainly: Anxiety Sensitivity Index-3 [56] (*n* = 10)*	7	4
Cognitive appraisals (10)	Threat interpretation, cognitive dissonance, estimates of peer drinking, body dissatisfaction, perceived drinking norms	*m* = 8 (mainly self-report, on word association test), e.g. Actual-Ideal Drinking Discrepancy	6	4
Dysfunctional beliefs and attitudes (13)	Dysfunctional attitudes, thin ideal internalization, alcohol attitudes and intentions, coupling beliefs, susceptibility cognitions, obsessive-or control-related beliefs, values	*m* = 12 (self-report), e.g. Dysfunctional Attitudes Scale	9	4
Expectancies (11)	Phobic event expectations, hope, positive expectations, alcohol expectancies	*m* = 8 (self-report), e.g. Beck Hopelessness Scale, Phobic Beliefs Scale	8	3
Metacognitive skills (6)	Mindfulness, self-exploration, insight, acceptance and diffusion, mentalization	*m* = 5 (self-report and observer-rated), e.g. Avoidance & Fusion Questionnaire – Youth	5	1
Negative thoughts (12)	Negative and positive cognitions, automatic negative thoughts, ruminative thinking style, catastrophizing, cognitive arousal and distortions	*m* = 10 (self-report), e.g. Children's Automatic Thought Scale	10	1
Problem solving (4)	Problem solving	*m* = 3 (self-report), e.g. Problem Solving Questionnaire	2	2
Self-esteem/self-efficacy (7)	Self-esteem, self-efficacy, self-image	*m* = 6 (self-report), e.g. Rosenberg Self-Esteem Scale	5	2
Trauma-related cognitions (7)	Trauma-related misappraisals, cognitions and memory characteristics	*m* = 2 (self-report), mainly: Children’s Post-Traumatic Cognitions Inventory [57] (*n* = 6)*	5	2
Family (54)			35	19
Family functioning (22)	Family accommodation, conflict, process, cohesion, communication, functioning, relationship quality, support and connectedness	*m* = 20 (self-report, different perspectives from adolescents and/ or parents), e.g. Parent–Adolescent Communication Scale	17	8
Parenting skills (19)	Parental discipline, monitoring, supervision, involvement and support, mindfulness, coping, positive and negative parenting	*m* = 18 (mainly self-report, few observer-rated by interview), e.g. Alabama Parenting Questionnaire	14	7
Parental burden (6)	Caregiver strain, parental mental health and post-traumatic beliefs	*m* = 4 (self-report), mainly: Symptom-Check-List [58] (*n* = 3)*	3	3
Parental resources (2)	Parental self-efficacy and competence	*m* = 2 (self-report), e.g. Parenting Stress Index	1	1
Behavioral (48)			34	14
Coping (12)	Coping strategies, secondary control coping, coping efficacy, pain coping,	*m* = 9 (mainly self-report), e.g. Children’s Coping Strategies Checklist	7	5
Motivation to change (7)	Change talk, motivation to change, reflect sustain talk, motivation for safety plan use	*m* = 6 (mainly observer or therapist-rated), e.g. Motivational Interviewing Skills Code	5	2
Engagement in positive behaviors (8)	Avoidance behavior, specific behavioral strategies, pleasant and physical activities, school Engagement, accomplishment motivation, safety plan use	*m* = 8 (only self-report and mainly created for the specific study)	6	2
Engagement in therapy activities (7)	Exposure compliance, involvement, out-of-session and in-session-engagement, resistance	*m* = 5 (only observer or therapist-rated), e.g. Child Involvement Rating Scale	6	1
Impulse control (8)	Protective behavioral strategies, behaviors problems, drink refusal, risk taking, restraint over eating	*m* = 7 (only self-report and mainly created for the specific study), e.g. Eysenck’s Risk-Taking Scale	7	1
Physical health behaviors (6)	Quality of sleep, healthy eating	*m* = 6 (self-report and objective), e,g. Hollland Sleep Disorder Questionnaire	3	3
Therapy-related (34)			19	15
Outcome-focused (12)	Change in symptoms (depression, anxiety, general), medication adherence	*m* = 11 (self-report, observer- and parent-rated), e.g. Schedule for Affective Disorders and Schizophrenia for School-Age Children	5	7
Alliance (10)	Alliance (child, therapist, agreement)	*m* = 6 (mainly self-report, few observer-rated), mainly: Therapeutic Alliance Scale for Children [59] (*n* = 3)*	6	4
Technique (8)	Use of open-ended questions, therapist language, empathy, reflections and adherence	*m* = 3 (mainly observer-rated), mainly: Motivational Interviewing Skills Code, Application for Coding Treatment Interactions [60] (*n* = 6)*	5	3
Treatment duration (4)	Mean duration, total number of treatment sessions, additional services	no standardized measure	3	1
Relational (23)			15	8
Peer influence (14)	Peer influence, support, discussion, relations, emotional bonding and conflict, deviant peer association	*m* = 12 (mainly self-report, few parent-report and observer-rated), e.g. Network of Relationships Inventory–Short Form	9	5
Interpersonal skills (7)	Interpersonal behavior, skills and competence, romantic and friend functioning, peer conflict	*m* = 6 (self-report and performance tests), e.g. Social Adjustment Scale	4	3
Attachment (2)	Attachment	*m* = 1 (self-report): Experience in Close Relationships	2	–
Emotional (13)			8	5
Recognition and expression of emotions (7)	Emotional self-awareness, exploration, expression and recognition, active inhibition of emotional expression, empathy	*m* = 7 (self-report, objective test, observer-rated, some developed for the specific study)	4	3
Emotion regulation (3)	Expressive suppression, stress regulation, distress tolerance, discomfort intolerance, emotion regulation	*m* = 4 (self-report), e.g. Discomfort Intolerance Scale	2	2
Loneliness (2)	Loneliness	*m* = 1 (self-report): Loneliness Scale	2	–

*When a measure was used at least in three different analysis, the measure is marked with an asterisk

For the cognitive mediators, nine different subtypes could be identified that were either closely connected to classic change theories from CBT-related therapies or related to third-wave or integrative therapies: (1) anxiety sensitivity, (2) cognitive appraisals, (3) dysfunctional beliefs and attitudes, (4) expectancies, (5) metacognitive skills, (6) negative thoughts, (7) problem solving, (8) self-esteem and self-efficacy, and (9) trauma-specific alterations of cognitions. 57 of the reported mediation tests of cognitive mediators (71%) were significant in contrast to 23 non-significant tests. Especially promising mediators with more than 65% significant results were identified in the domain of changes in negative thoughts (10 significant vs. one non-significant results), changes in metacognitive skills (five significant vs. one non-significant results) as well as dysfunctional beliefs and attitudes (nine significant vs. four non-significant results). Expectancies, self-efficacy/esteem as well as trauma-related cognitions also had a majority of significant mediation results. Anxiety-sensitivity as well as cognitive appraisals were below but close to the 65% threshold of number of significant results across all studies. Only problem-solving had inconclusive results over four studies. While cognitive mediators seemed mostly CBT-specific, no age specific constructs could be identified that were only used in this age group. However, all cognitive measures used were adapted to age-specific cognitions.

The relatively high number of family-related mediators with *k* = 54 is most likely linked to the frequency of family therapy settings in treating adolescents as well as the 16 studies on systemic therapy that were included in the review. Mediators from this group were subdivided into four specific sub-categories including (1) family functioning, (2) parental resources, (3) parenting skills and (4) parental burden. The ratio between significant and non-significant mediators is in favor of family-related changes to explain outcome, but the evidence is less robust than for cognitive mediators with 35 significant vs. 19 non-significant results (64.8% significant). Within the category, family functioning and parenting skills can be regarded as the most promising mediators since changes in parenting skills had 66.7% significant mediation results along with family functioning that had 68% significant results. Parental burden and parental resources had equal numbers of significant and non-significant mediation results and thus their evidence base is classified as inconclusive. In general, family-related mediators could be regarded as setting- and age-specific mediators that are rarely if ever used in adult psychotherapy mediation research. Interestingly, family mediators focused mainly on parents and thus measures were mostly completed by parents.

With regards to behavioral mediators, 6 distinct sub-categories were identified that were related to (1) coping, (2) motivation to change, (3) engagement in positive general behavior, (4) engagement in therapy activities, (5) impulse control, and (6) behavior promoting physical health. Overall, behavioral mediators turned out to have highly convincing evidence with 71% of the studies showing significant mediation effects. The most promising in the studies were changes in successful engagement in therapy activities as well as increased impulse control (mainly in studies on substance abuse disorders or eating disorders). Also, the engagement in generally positive behaviors outside therapy appeared to be very convincing on the one hand and a higher motivation to change over the course of treatment on the other hand (mainly assessed in humanistic approaches such as Motivational Interviewing interventions). Less robust with 58% significant results was coping behavior. The only inconclusive results appeared in the sub-category changes in physical health behaviors, such as sleep hygiene and health, as an equal number of mediators were found to be significant and non-significant in six different analyses.

We found 34 therapy-related mediators that were divided into four categories: (1) outcome-focused (in terms of typically being investigated as ends in themselves, e.g., changes in symptoms), (2) treatment duration, (3) therapeutic alliance, and (4) therapist techniques. Aside from therapeutic alliance, all mediators in this category differed from the classic understanding of a mediator in psychotherapy, i.e., a variable that is changing through a certain technique or intervention which later on is followed by a change in symptoms. Nevertheless, a change in symptoms, for instance, could possibly change another outcome, and thus be considered a mediator. However, using a traditional outcome variable such as symptoms as a mediator was the only sub-category in which more non-significant than significant results were reported. With regards to therapist technique, 62.5% of the mediation analysis were significant. Similarly, 60% of the alliance mediators were reported significant. Only three studies investigated treatment duration, none of which used a standardized measure. Therapy-related mediators generally showed a rather poor rate of significant results of only 56%.

Relational mediators were investigated in a total of 23 tests. These were divided into three different sub-categories: (1) how patients were influenced by their peers during therapy, (2) whether their interpersonal skills improved, and (3) whether their attachment style changed. In total, significant results were found 65% of the time. Peer influence can be regarded as an age-specific and promising mediator with 64% significant results, whereas interpersonal skills were found almost as often significant as non-significant. Changes in attachment had 100% significant mediation effects, but this was based on only two studies and thus needs replication.

Finally, emotional mediators were used in 20 analyses and were classified into three sub-categories: (1) emotion recognition and expression, (2) emotion regulation, as well as (3) loneliness. Sixty-two percent of the mediation tests were significant, leading also to rather inconclusive evidence for the role of emotional mediators in the psychosocial treatment of adolescents. However, this has to be interpreted in relation to the range of different assessment types (from self-report measures, observer-rated measures to objective tests) which was highest within this category.

**Fig. 2:**
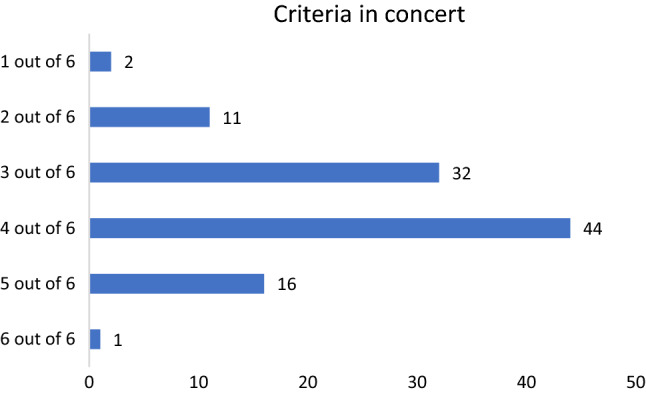
The study quality criteria in concert: number of studies per number of criteria met

Following the study quality assessment recommendations by Kazdin [13] and Lemmens et al. [28], all 106 studies were rated for each of the six criteria (compare Table 4  and Fig. 2). As we only included RCTs, 100% of studies fulfilled the first criterion. Eighty-nine studies tested the mediator in the experimental and the control group, 79 studies had a sufficient sample size, 68 studies assessed multiple mediators and 39 fulfilled the temporality criterion, i.e., assessed the mediator at least three times during the trial. Finally, four studies aimed at experimentally manipulating the mediator although none of the studies did this using different levels of the mediator but had essentially two levels. Only one study (i.e., [61]) fulfilled all six criteria outlined by Kazdin [13] for rigorously evaluating and identifying potential mediators.

**Table 4:** Number (%) of studies meeting requirements for process research (*n* = 106)

Requirement	*n* studies (%)
RCT, yes (%)	106 (100)
Control group, yes (%)	89 (84.0)
Sample size per condition ≥ 40, yes, *n* (%)	79 (74.5)
Multiple mediators, yes, *n* (%)	68 (64.2)
Assessment of temporality, yes, *n* (%)	39 (36.8)
Manipulation of mediator/experiment, yes, *n* (%)	4 (3.8)

Using the BESRS (using the ROB-rating score in combination with the Kazdin criteria), we classified the 106 studies into *k* = 12 good study quality, *k* = 26 satisfactory study quality and *k* = 68 unsatisfactory study quality. In the following step of the BESRS, the evidence base of different mediator types was assessed according to diagnosis using the overall study quality, number of RCTs, as well as percentage of significant mediators (compare Table 5). First, there was no strong evidence for any mediator type in any diagnosis group. Furthermore, the evidence base for different mediator types differed notably across diagnoses. For anxiety disorders, moderate evidence emerged only for cognitive mediators, and the same applied to PTSD (classified in the prior DSM-IV also among the anxiety disorders). However, the majority of these studies investigating cognitive mediators in anxiety disorder treatments were considered as unsatisfactory quality and none were of good quality. Depression presented a considerably different picture, with moderate evidence for almost all mediator types, i.e., behavioral, cognitive, family, relational, and those classified as ‘therapy-related’. Further, each of these mediator types was supported by at least one good-quality study. For externalizing disorders, the most frequently studied mediators, i.e., family and relational mediators, also received moderate evidence and were each supported by at least one good-quality study, whereas other mediator types remained virtually unstudied. Finally, several mediator types were investigated in substance abuse disorders and received moderate evidence, i.e., behavioral, cognitive, family, and those classified as ‘therapy-related’, with all having at least one good-quality or several satisfactory-quality studies. For almost all diagnoses, however, the number of studies on particular mediator types was notably too small for drawing stronger conclusions.

**Table 5:** Summary of evidence for the type of potential mediators identified in psychological treatments for anxiety, depression, externalizing disorders, substance use disorders and PTSD in adolescents

	Examined studies (examined mediators)^a^	Significant studies^b^			Global evidence^c^
Anxiety		Good	Satisfactory	Unsatisfactory	
Behavioral	6 (10)	–	2 (3)	3	Moderate
Cognitive	11 (19)	–	2 (4)	7 (10)	Moderate
Emotional	3 (4)	–	–	1 (2)	Insufficient
Family	2 (3)	–	1 (2)	1	Insufficient
Relational	3 (5)	–	–	2	Insufficient
Therapy-related	–	–	–	–	Insufficient
Depression					
Behavioral	3	1	–	2	Moderate
Cognitive	10 (11)	1	3	6	Moderate
Emotional	2 (3)	–	1	1 (2)	Insufficient
Family	6 (7)	2	–	3	Moderate
Relational	5 (8)	1 (2)	1	2	Moderate
Therapy-related	3 (4)	1	–	2	Moderate
Externalizing disorders					
Behavioral	2 (3)	–	–	1 (2)	Insufficient
Cognitive	1 (3)	–	–	1 (3)	Insufficient
Emotional	1 (2)	–	–	–	Insufficient
Family	9 (22)	3 (6)	2 (4)	3 (5)	Moderate
Relational	8 (10)	1	1	4	Moderate
Therapy-related	3	–	1	–	Insufficient
Substance use					
Behavioral	13 (16)	1	5 (7)	6	Moderate
Cognitive	16 (22)	–	3	8 (12)	Moderate
Emotional	–	–	–	–	Insufficient
Family	4 (5)	1	1 (2)	1	Moderate
Relational	4 (5)	–	–	2	Insufficient
Therapy-related	7 (10)	1	2 (3)	2 (3)	Moderate
PTSD					
Behavioral	1 (2)	–	1	–	Insufficient
Cognitive	7 (8)	–	1	5	Moderate
Emotional	–	–	–	–	Insufficient
Family	1	–	–	1	Insufficient
Relational	–	–	–	–	Insufficient
Therapy-related	1	–	–	–	Insufficient

^a^As some studies investigated more than one mediator in the particular category, the number in parentheses indicates the overall number of specific mediators investigated in the particular category

^b^Good: If the total score for the risk of bias in an RCT was five or six, and the study met five or six of Kazdin’s criteria. Satisfactory: If the total score for the risk of bias in an RCT was five or six, and the study met four of Kazdin’s criteria. Unsatisfactory: If the total score for the risk of bias was below five and the study met less than four of Kazdin’s criteria

^c^Strong: If 65% of studies found significant mediators in one category across at least three RCTs and the quality of these RCTs was rated good or satisfactory. Moderate: If 65% of studies found significant mediators in one category across at least three RCTs and the quality of these RCTs was rated good, satisfactory or unsatisfactory. Insufficient: If less than 65% of studies found significant mediators in one category in less than three RCTs and the quality of these RCTs was rated unsatisfactory

## Discussion

As far as we know, this is the first systematic review assessing mediators of psychotherapeutic changes in adolescents across treatments and diagnoses. By virtue of our transtheoretical and transdiagnostic approach, we extended a broader net than prior reviews to include a range of different therapeutic approaches, and found that mediator studies have involved mainly CBT, Systemic Therapy, Humanistic Therapy and Educational approaches. Regarding knowledge on therapeutic change mechanisms for adolescents, we found there is a striking lack of RCT mediator studies for IPT, third-wave therapies and above all—in view of their prevalence [62]—for psychodynamic therapies. All in all, 252 results on mediator-analyses in 106 studies were identified.

Concerning the question of which mediators have been studied in psychotherapy RCTs with adolescents, the biggest group of mediators were from the cognitive field, followed by family-related, behavioral, therapy-related, relational, and emotional mediators, respectively. Given the generally accepted importance of improved emotion regulation for successful therapies (e.g. [63, 64]), and how critical emotion regulation skills become during the sometimes tumultuous years of adolescence [65], the relatively small percentage of studies investigating emotional mediators is surprising. However, this may partly reflect methodological challenges, e.g., in reliably measuring emotional processes and separating change in them from change in ultimate outcomes, e.g., reduction in depressive symptoms. Overall, the 252 mediators were assessed with 181 different measures which comprised mainly self-report measures. This strongly suggests that there is little consensus on the central change mechanisms or related mediator measures between or *even within* different therapeutic approaches, irrespective of diagnoses. Thus, to create an orienting overview for future studies, mediators were subcategorized within the broader domains to identify putative transdiagnostic and transtheoretical change mechanisms that are supported by research. Within each category some mediators were identified to be promising for future investigations: changes in negative thoughts, dysfunctional beliefs and metacognitive skills in the cognitive category; family functioning and parenting skills among family-related mediators; and successful engagement in therapy activities as well as increased impulse control in the behavioral mediator category. This is partly confirming former reviews on adolescents that reported significant mediation results for changes in negative thoughts, family functioning, social engagement as well as parenting [31, 32]. Results on relational, therapy-related, and emotional mediators were inconclusive due to fewer studies, as well as a higher percentage of insignificant results, although peer-influence appears a promising mediator.

With regard to our second research question of whether there are age-, disorder- and treatment-specific mediators, we conclude that, in comparison to research in adult samples, age-specific mediators with age-adjusted measures have been studied. More importantly, some promising findings of significant mediators emerged especially in the domains of family-related changes (e.g., family functioning and parenting), specific adolescent behaviors (e.g., engagement in therapy) as well as relational changes (e.g., peer-relations). Further findings indicate that the studied constructs of cognitive mediators do not greatly differ from those in research on adult psychotherapy, although appropriately age-adjusted measures have been used with adolescent populations. Various disorders have been the target of mediator studies up until now, mainly focusing on anxiety, depression, externalizing disorders, trauma and substance abuse. Fewer studies have focused on OCD and eating disorders, singular studies have addressed other diagnoses, and no study in our review focused on change mechanisms in personality disorders among adolescents. Certain types of mediators have been investigated specifically in the context of certain diagnoses (e.g., phobic beliefs, post-traumatic cognitions, or antisocial peer influence). However, the majority of mediators have been studied across different diagnoses and could be characterized as non disorder-specific but rather relevant to common change processes.

The research evidence for particular mediators within the main diagnoses ranged between moderate and insufficient. None of the mediator categories (cognitive, family-related, behavioral, relational, therapy-related and emotional) could be classified as having strong evidence. No sufficient evidence was found for emotional mediators as these were rarely examined. Moderate evidence for the other categories was found in studies on anxiety for behavioral and cognitive mediators, in studies on externalizing disorders for family and relational mediators, in studies on substance abuse for all except relational mediators, in studies on trauma for cognitive mediators, and in studies on depression for all mediator categories. Perhaps unsurprisingly, we also found that certain therapeutic approaches were investigated more often in certain diagnoses: more CBT studies in anxiety and trauma, more humanistic (typically motivational interviewing) studies in substance abuse, and more systemic therapy studies in externalizing disorders. Given the tendency to mainly test treatment-specific change mechanisms without considering other possible change theories, the findings on posited diagnosis-specific mediators are unavoidably, if understandably, confounded by researcher bias. By investigating mainly mediators that are in line with specific therapeutic models, the commonly used research designs follow a logic of verifying rather than falsifying theoretical assumptions [66]. Thus, at present, the research appears insufficient for objectively and reliably identifying disorder-specific mediators.

More generally also, we note that only nine of 106 (8.8%) studies could be classified as having both relatively low risk of bias in design and proper rigor in testing mediation models. On the contrary, the majority of studies (*k* = 69) (67.6%) needed to be classified as being of poor quality, due to unclear reporting or limitations in one or both aspects. This highlights the importance of thoroughly reporting all aspects of study design for future mediation studies. More specifically, in terms of Kazdin’s criteria—i.e., recommendations about statistical power, the temporal criterion, reliable measures and tests, specificity, and the experimental manipulation of the mediator—virtually all studies had at least some shortcomings. With regards to statistical power, as the mediation tests comprised mostly secondary analysis, no study reported a power analysis for mediation testing in preparation for the trial. Yet, at least in the broad sense of having a group larger than 40 participants, fortunately almost 74% of the reviewed studies appeared sufficiently large for investigating mediation (cp., [28]). However, 64.7% of the studies did violate the temporal criterion, which has been noted as a concern before [21, 67, 68]. Perhaps the most alarming finding of this comprehensive review was the exceedingly large number of measures used to assess mediators; indeed, each mediator measure was only seldom employed in more than one or two studies. This heterogeneity should be kept in mind as it undermines construct validity and findings of significance within mediator categories should be therefore interpreted with caution. Furthermore, almost all of the used measures were self-reports which shows a methodologically limited perspective, with very few studies using observer-rated or objective measures (e.g., computer task).

With regard to statistical tests of mediation, our review identified 14 different methodologies; the majority of studies used bootstrapping or SEM methods. However, as far as we could make out, 33 studies did not report a specific estimation method for the indirect effect of the mediator on outcome. As there is currently no generally accepted gold standard for mediation analysis, these were all treated similarly in our BESRS rating system. Future advances in establishing such standards might nevertheless also provide more nuance to the current findings and there is a high need for consensus guidelines in reporting and planning of mediation studies overall [33, 69]. In our review, there was only a small number of studies showing specific and robust associations between mediator and outcome in the intervention group only (i.e. [70–74])—although in these studies the overall evidence was slightly less strong, in terms of either experimentally manipulating the mediator or assessing alternative mediators. The main limitation shared by virtually all the studies in terms of study design was a lack of clear experimental manipulation of the mediator [75]. The same limitation was also identified by Lemmens and colleagues [28] in their review of studies on mechanisms of change in adulthood depression. They speculated that a possible reason might be that treatments in which only one isolated mediator is manipulated while keeping everything else constant would likely lack external validity. Considering the lack of experimental manipulation of the mediator in most study designs, it seems important to mention also a few clinically relevant studies whose designs seemed commendable for attempts to specifically influence the mediator and assess its impact on the outcome, such as the studies on at-risk adolescents for anxiety [76] or eating disorders [61]. These study designs thus show that, with sufficient creativity, disentangling plausible mediators can also be achieved in ecologically valid investigations of clinically relevant phenomena. The limited number of studies manipulating the mediator directly does not mean that such studies should not be attempted or are not possible. Indeed, recent literature has seen discussion of various alternatives in both experimental study design and statistical methodology to enhance the inferring of causality in mediation studies [75, 77, 78]. As noted already long ago [79], but discussed more recently also by Kazdin [80] and others (e.g., [81, 82]), causality can reasonably be inferred even in the absence of randomization—and, in some cases, has to be. Furthermore, multiple alternative designs and methods can be useful in shedding light on mechanisms of change, such as single-case experimental studies [80] and qualitative work embedded in RCTs (e.g., [83]).

The studies included in this review were primarily conducted in the western industrialized world which constitutes a limitation in terms of representativity. And while there is an excellent gender balance, studies failed to report if results differed with respect to gender, preventing conclusions on gender-specific mediators for adolescents.

Given the considerable heterogeneity between studies (most notably the multitude of mediator constructs and measures) and no agreed upon effect size in mediation studies, it was not feasible nor meaningful to estimate aggregated effect sizes for the identified mediators which is an important task for future mechanism research. Thus, no funnel plot test for evaluating publication bias was possible. While we can state that a third of the mediation effects were published with non-significant results, the extent of the file-drawer problem—i.e., non-significant results remaining unpublished—is difficult to evaluate.

Despite multiple limitations, our results align with other recent reviews [28, 31, 33], in calling for further investigation and greater methodological rigor in order to identify specific mediators leading to positive change in psychotherapy for adolescents. This identification of significant mediators will enable more reliable conclusions about the underlying mechanisms of change and the possible theoretical models supporting different forms of psychotherapy.

The findings of this review also allow for some tentative and preliminary observations to be considered in developing future treatment models. First, multiple mediators across mediator categories emerged as significant in the treatment of depression, across theoretical treatment models (Table 4). This fits well with the fact that numerous psychotherapeutic approaches, aiming to effect change through somewhat different pathways, have been found to be equally effective for treating depression [84]. The practical implication would be that these different approaches might benefit from further investigating mediators that are not restricted to their particular change theory, in order to clarify the prevalent specific vs. common factors debate [14]. Such future studies could incorporate both the study of transtheoretical mediators such as the ones identified in this review, as well as transdiagnostic mediators pertaining to the particular developmental tasks and challenges of adolescence (e.g., emotion regulation, self-image, peer influence, etc.).

In terms of identifying promising transdiagnostic mediators, most support was found for behavioral and cognitive mediators, followed by family-related mediators. That is, behavioral, cognitive and family-related mediators seemed to have a significant effect on the outcome in psychological interventions across diagnostic categories. While the findings of behavioral and cognitive mediators was perhaps not a surprise given the large number of studies on cognitive-behavioral treatments, the finding on family-related mediators does underline the importance of family and parental involvement in the treatment of adolescents. Accordingly, irrespective of diagnosis, future studies on the psychological treatment of adolescents might include measures of family-related mediators to clarify how families may facilitate or hinder adolescent mental health and recovery from mental illness. Network analyses may also prove useful in helping to elucidate how changes in one domain (e.g. family functioning) may affect changes in another domain (e.g. behavioral changes) or vice-versa [85].

Efforts to identify common, transdiagnostic factors that lead to positive therapeutic change are in line with a dimensional approach to diagnosis and psychopathology, which has been gaining attention (e.g. Caspi et al. [86]), as categorical approaches such as the DSM have come under severe scrutiny and criticism (e.g. [87]). In addition to not finding enough support for diagnosis-specific mediators, our results also indicate little evidence for treatment-specific mediators. While Kazdin’s criteria for mediators imply that true mediators should be treatment-specific, our results indicated that mediators were often significant both in the experimental treatment condition and in the control condition. These findings suggest that the search for specific mediators of specific psychotherapies—as they are currently defined—may often yield little, as potent curative elements may plausibly be shared across treatment and even control conditions [88].

Thus, in line with previous reviews, we advocate that both specific and non-specific mediators and processes should be investigated to validate as well as falsify particular theories of change, including non-psychological such as biological mediators. While the lack of biological mediators is not limited to adolescence research, with a recent review on brain-related functional mediators in adult psychotherapy failing to identify any studies on them [89], studying biological change mechanisms in adolescents’ psychotherapy could be particularly informative as adolescence is a time of rapid and pervasive biological maturation. This would also call for increased interdisciplinary collaboration and openness towards models of change on various interacting explanatory levels as opposed to those operating only on the psychological level, let alone from a particular conceptual model.

To summarize some conclusions for further research, in line with similar initiatives, we see that as each therapeutic approach may address numerous change mechanisms [14], and diagnostic categories may be at the same time too narrow and too broad when looking at change mechanisms [90, 91], a transtheoretical and transdiagnostic turn to investigating more concrete and specific mechanisms of change across diagnostic categories is called for. A similar approach has already been advocated e.g., by the National Institute of Mental Health’s Research Domain Criteria [92], recommending a dimensional rather than categorical approach to understanding mental illness and its early determinants on various levels, from the neural to the behavioral. Likewise, modular therapy approaches (e.g., [93]) with randomization at the level of modules addressing specific change mechanisms may offer better opportunities for identifying change mechanisms than comparing broad therapeutic orientations Another research initiative on specifically behavioral health problems (e.g., smoking, alcohol, substance abuse, poor diet, lack of exercise) has underlined the need to establish a core set of measures going beyond treatment-specific change theories and gauging various developmental challenges and observational perspectives [94]. From our point of view, this core set should also include biological change factors such as heart-rate-variability, cortisol, and other stress-related constructs.

## Conclusions

To sum up our key findings, by not being restricted to specific theoretical models or specific diagnoses, our review identified general support for the existence of age-specific mediators pertaining to adolescents (i.e., the significance of family and peer-related mediators). This important finding suggests that rather than attempting to transfer new models from adult psychotherapy to treating adolescents, therapeutic outcomes might be better enhanced by focusing on developing sophisticated age-specific models of therapeutic change. However, research on change mechanisms investigated has rarely addressed the specific developmental challenges in adolescence. Our review suggests that adolescent models of psychotherapy would do well to adopt a developmental perspective which takes into account the many ways in which adolescent psychotherapy differs from the psychotherapy of adults, including the factors and mechanisms that may influence therapeutic change.

By extending a broad net across treatment models and diagnosis, this review has investigated and documented all the different kinds of mediators that have been studied to date in adolescent psychotherapy. This inclusiveness has been fruitful in recognizing that different treatment models may be identifying similar factors at work that lead to positive therapeutic outcomes. There are great benefits to taking a transtheoretical and transdiagnostic approach, as researchers can learn from different models about the variety of changes that can be observed in the process of psychotherapy. Both the field of psychotherapy research and the populations we serve stand to gain from the willingness of researchers to step outside model-specific comfort zones and include in their field of inquiry less familiar ways of thinking about how therapeutic change transpires.

## Supplementary Information

Below is the link to the electronic supplementary material.

Supplementary file1 (DOCX 29 KB)
