# Supplementary material for: Mediators of outcome in adolescent psychotherapy and their implications for theories and mechanisms of change: a systematic review
Source: Eur Child Adolesc Psychiatry. 2023 Mar 15;33(10):3321–58. doi: 10.1007/s00787-023-02186-9 (PMC11564358; doi:10.1007/s00787-023-02186-9)
Supplement: Supplementary file 1 — Supplementary file1 (DOCX 29 KB) [file 787_2023_2186_MOESM1_ESM.docx]

# References of included studies

Alfano, C. A., Pina, A. A., Villalta, I. K., Beidel, D. C., Ammerman, R. T., & Crosby, L. E. (2009). Mediators and moderators of outcome in the behavioral treatment of childhood social phobia. *Journal of the American Academy of Child and Adolescent Psychiatry*, *48*(9), 945–953. https://doi.org/10.1097/CHI.0b013e3181af8216

Allan, N. P., Short, N. A., Albanese, B. J., Keough, M. E., & Schmidt, N. B. (2015). Direct and Mediating Effects of an Anxiety Sensitivity Intervention on Posttraumatic Stress Disorder Symptoms in Trauma-Exposed Individuals. *Cognitive Behaviour Therapy*, *44*(6), 512–524. https://doi.org/10.1080/16506073.2015.1075227

Asarnow, J., Berk, M., Bedics, J., Adrian, M., Gallop, R., & Cohen, J. et al. (2021). Dialectical Behavior Therapy for Suicidal Self-Harming Youth: Emotion Regulation, Mechanisms, and Mediators. *Journal Of The American Academy Of Child &Amp; Adolescent Psychiatry*, *60*(9), 1105-1115.e4. doi: 10.1016/j.jaac.2021.01.016

Bakhshaie, J., Geller, D. A., Wilhelm, S., McGuire, J. F., Small, B. J., Cepeda, S. L., Schneider, S. C., Murphy, T. K., Porth, R., & Storch, E. A. (2020). Temporal precedence of the change in obsessive-compulsive symptoms and change in depressive symptoms during exposure and response prevention for pediatric obsessive-compulsive disorders. *Behaviour Research and Therapy*, *133*, 103697. https://doi.org/10.1016/j.brat.2020.103697

Barnett, E., Moyers, T. B., Sussman, S., Smith, C., Rohrbach, L. A., Sun, P., & Spruijt-Metz, D. (2014). From counselor skill to decreased marijuana use: Does change talk matter? *Journal of Substance Abuse Treatment*, *46*(4), 498–505. https://doi.org/10.1016/j.jsat.2013.11.004

Barnett, N. P., Murphy, J. G., Colby, S. M., & Monti, P. M. (2007). Efficacy of counselor vs. Computer-delivered intervention with mandated college students. *Addictive Behaviors*, *32*(11), 2529–2548. https://doi.org/10.1016/j.addbeh.2007.06.017

Black, J. J., Tran, G. Q., Goldsmith, A. A., Thompson, R. D., Smith, J. P., & Welge, J. A. (2012). Alcohol expectancies and social self-efficacy as mediators of differential intervention outcomes for college hazardous drinkers with social anxiety. *Addictive Behaviors*, *37*(3), 248–255. https://doi.org/10.1016/j.addbeh.2011.10.004

Blake, M., Schwartz, O., Waloszek, J. M., Raniti, M., Simmons, J. G., Murray, G., Blake, L., Dahl, R. E., Bootzin, R., McMakin, D. L., Dudgeon, P., Trinder, J., & Allen, N. B. (2017). The SENSE Study: Treatment Mechanisms of a Cognitive Behavioral and Mindfulness-Based Group Sleep Improvement Intervention for At-Risk Adolescents. *Sleep*, *40*(6). https://doi.org/10.1093/sleep/zsx061

Blake, M. J., Snoep, L., Raniti, M., Schwartz, O., Waloszek, J. M., Simmons, J. G., Murray, G., Blake, L., Landau, E. R., Dahl, R. E., Bootzin, R., McMakin, D. L., Dudgeon, P., Trinder, J., & Allen, N. B. (2017). A cognitive-behavioral and mindfulness-based group sleep intervention improves behavior problems in at-risk adolescents by improving perceived sleep quality. *Behaviour Research and Therapy*, *99*, 147–156. https://doi.org/10.1016/j.brat.2017.10.006

Bonnert, M., Olén, O., Bjureberg, J., Lalouni, M., Hedman-Lagerlöf, E., Serlachius, E., & Ljótsson, B. (2018). The role of avoidance behavior in the treatment of adolescents with irritable bowel syndrome: A mediation analysis. *Behaviour Research and Therapy*, *105*, 27–35. https://doi.org/10.1016/j.brat.2018.03.006

Borduin, C., Quetsch, L., Johnides, B., & Dopp, A. (2021). Long-term effects of multisystemic therapy for problem sexual behaviors: A 24.9-year follow-up to a randomized clinical trial. *Journal Of Consulting And Clinical Psychology*, *89*(5), 393-405. doi: 10.1037/ccp0000646

Borsari, B [Brian], Apodaca, T. R., Jackson, K. M., Mastroleo, N. R., Magill, M., Barnett, N. P., & Carey, K. B [Kate B.] (2015). In-session processes of brief motivational interventions in two trials with mandated college students. *Journal of Consulting and Clinical Psychology*, *83*(1), 56–67. https://doi.org/10.1037/a0037635

Borsari, B [B.], & Carey, K. B [K. B.] (2000). Effects of a brief motivational intervention with college student drinkers. *Journal of Consulting and Clinical Psychology*, *68*(4), 728–733.

Botvin, G. J., Schinke, S. P., Epstein, J. A., Diaz, T., & Botvin, E. M. (1995). Effectiveness of culturally focused and generic skills training approaches to alcohol and drug abuse prevention among minority adolescents: Two-year follow-up results. *Psychology of Addictive Behaviors*, *9*(3), 183–194. https://doi.org/10.1037/0893-164X.9.3.183

Boyer, B., MacKay, K. J., McLeod, B. D., & van der Oord, S. (2018). Comparing Alliance in Two Cognitive-Behavioural Therapies for Adolescents With ADHD Using a Randomized Controlled Trial. *Behavior Therapy*, *49*(5), 781–795. https://doi.org/10.1016/j.beth.2018.01.003

Brent, D. A [D. A.], Kolko, D. J., Birmaher, B [B.], Baugher, M., Bridge, J., Roth, C., & Holder, D. (1998). Predictors of treatment efficacy in a clinical trial of three psychosocial treatments for adolescent depression. *Journal of the American Academy of Child and Adolescent Psychiatry*, *37*(9), 906–914. https://doi.org/10.1097/00004583-199809000-00010

Brody, G. H., Yu, T., Chen, Y., Kogan, S. M., & Smith, K. (2012). The Adults in the Making program: Long-term protective stabilizing effects on alcohol use and substance use problems for rural African American emerging adults. *Journal of Consulting and Clinical Psychology*, *80*(1), 17–28. https://doi.org/10.1037/a0026592

Bruin, E. J. de, Bögels, S. M., Oort, F. J., & Meijer, A. M. (2018). Improvements of adolescent psychopathology after insomnia treatment: Results from a randomized controlled trial over 1 year. *Journal of Child Psychology and Psychiatry, and Allied Disciplines*, *59*(5), 509–522. https://doi.org/10.1111/jcpp.12834

Brunwasser, S. M., Freres, D. R., & Gillham, J. E. (2018). Youth Cognitive-Behavioral Depression Prevention: Testing Theory in a Randomized Controlled Trial. *Cognitive Therapy and Research*, *42*(4), 468–482.

Carey, K. B [Kate B.], Walsh, J. L., Merrill, J. E., Lust, S. A., Reid, A. E., Scott-Sheldon, L. A. J., Kalichman, S. C., & Carey, M. P. (2018). Using e-mail boosters to maintain change after brief alcohol interventions for mandated college students: A randomized controlled trial. *Journal of Consulting and Clinical Psychology*, *86*(9), 787–798. https://doi.org/10.1037/ccp0000339

Chaplin, T., Mauro, K., Curby, T., Niehaus, C., Fischer, S., & Turpyn, C. et al. (2021). Effects of A Parenting-Focused Mindfulness Intervention on Adolescent Substance Use and Psychopathology: A Randomized Controlled Trial. *Research On Child And Adolescent Psychopathology*, *49*(7), 861-875. doi: 10.1007/s10802-021-00782-4

Chen, Y., Yu, T., & Brody, G. H. (2017). Parenting Intervention at Age 11 and Cotinine Levels at Age 20 Among African American Youth. *Pediatrics*, *140*(1). https://doi.org/10.1542/peds.2016-4162

Chu, B. C., & Kendall, P. C [Philip C.] (2004). Positive association of child involvement and treatment outcome within a manual-based cognitive-behavioral treatment for children with anxiety. *Journal of Consulting and Clinical Psychology*, *72*(5), 821–829. https://doi.org/10.1037/0022-006X.72.5.821

Compas, B. E., Champion, J. E., Forehand, R., Cole, D. A., Reeslund, K. L., Fear, J., Hardcastle, E. J., Keller, G., Rakow, A., Garai, E., Merchant, M. J., & Roberts, L. (2010). Coping and parenting: Mediators of 12-month outcomes of a family group cognitive-behavioral preventive intervention with families of depressed parents. *Journal of Consulting and Clinical Psychology*, *78*(5), 623–634. https://doi.org/10.1037/a0020459

Czyz, E. K., King, C. A., & Biermann, B. J. (2019). Motivational Interviewing-Enhanced Safety Planning for Adolescents at High Suicide Risk: A Pilot Randomized Controlled Trial. *Journal of Clinical Child and Adolescent Psychology : The Official Journal for the Society of Clinical Child and Adolescent Psychology, American Psychological Association, Division 53*, *48*(2), 250–262. https://doi.org/10.1080/15374416.2018.1496442

Dadds, M. R [Mark Richard], Cauchi, A. J., Wimalaweera, S., Hawes, D. J., & Brennan, J. (2012). Outcomes, moderators, and mediators of empathic-emotion recognition training for complex conduct problems in childhood. *Psychiatry Research*, *199*(3), 201–207. https://doi.org/10.1016/j.psychres.2012.04.033

D'Amico, E. J., Houck, J. M., Hunter, S. B., Miles, J. N. V., Osilla, K. C., & Ewing, B. A. (2015). Group motivational interviewing for adolescents: Change talk and alcohol and marijuana outcomes. *Journal of Consulting and Clinical Psychology*, *83*(1), 68–80. https://doi.org/10.1037/a0038155

Deković, M., Asscher, J. J., Manders, W. A., Prins, P. J. M [Pier J. M.], & van der Laan, P. (2012). Within-intervention change: Mediators of intervention effects during multisystemic therapy. *Journal of Consulting and Clinical Psychology*, *80*(4), 574–587. https://doi.org/10.1037/a0028482

Diamond, G. S., Liddle, H. A., Wintersteen, M. B., Dennis, M. L., Godley, S. H., & Tims, F. (2006). Early therapeutic alliance as a predictor of treatment outcome for adolescent cannabis users in outpatient treatment. *The American Journal on Addictions*, *15 Suppl 1*, 26–33. https://doi.org/10.1080/10550490601003664

Dietz, L. J., Marshal, M. P., Burton, C. M., Bridge, J. A., Birmaher, B [Boris], Kolko, D., Duffy, J. N., & Brent, D. A [David A.] (2014). Social problem solving among depressed adolescents is enhanced by structured psychotherapies. *Journal of Consulting and Clinical Psychology*, *82*(2), 202–211. https://doi.org/10.1037/a0035718

Doumas, D. M., McKinley, L. L., & Book, P. (2009). Evaluation of two Web-based alcohol interventions for mandated college students. *Journal of Substance Abuse Treatment*, *36*(1), 65–74. https://doi.org/10.1016/j.jsat.2008.05.009

Dunn, M. E., Fried-Somerstein, A., Flori, J. N., Hall, T. V., & Dvorak, R. D. (2020). Reducing alcohol use in mandated college students: A comparison of a Brief Motivational Intervention (BMI) and the Expectancy Challenge Alcohol Literacy Curriculum (ECALC). *Experimental and Clinical Psychopharmacology*, *28*(1), 87–98. https://doi.org/10.1037/pha0000290

Eddy, J. M., & Chamberlain, P. (2000). Family management and deviant peer association as mediators of the impact of treatment condition on youth antisocial behavior. *Journal of Consulting and Clinical Psychology*, *68*(5), 857–863. https://doi.org/10.1037/0022-006X.68.5.857

Fjermestad, K. W., Føreland, Ø., Oppedal, S. B., Sørensen, J. S., Vognild, Y. H., Gjestad, R., Öst, L.‑G., Bjaastad, J. F., Shirk, S. S., & Wergeland, G. J. (2021). Therapist Alliance-Building Behaviors, Alliance, and Outcomes in Cognitive Behavioral Treatment for Youth Anxiety Disorders. *Journal of Clinical Child and Adolescent Psychology : The Official Journal for the Society of Clinical Child and Adolescent Psychology, American Psychological Association, Division 53*, *50*(2), 229–242. https://doi.org/10.1080/15374416.2019.1683850

Fjermestad, K. W., Lerner, M. D., McLeod, B. D., Wergeland, G. J. H., Heiervang, E. R., Silverman, W. K., Öst, L.‑G., Los Reyes, A. de, Havik, O. E., & Haugland, B. S. M. (2016). Therapist-youth agreement on alliance change predicts long-term outcome in CBT for anxiety disorders. *Journal of Child Psychology and Psychiatry, and Allied Disciplines*, *57*(5), 625–632. https://doi.org/10.1111/jcpp.12485

Forsberg, S., Darcy, A., Bryson, S. W., Arnow, K. D., Datta, N., Le Grange, D., & Lock, J. (2017). Psychological symptoms among parents of adolescents with anorexia nervosa: a descriptive examination of their presence and role in treatment outcome. *Journal of Family Therapy*, *39*(4), 514–536. https://doi.org/10.1111/1467-6427.12088

Fosco, G. M., van Ryzin, M. J., Connell, A. M., & Stormshak, E. A. (2016). Preventing adolescent depression with the family check-up: Examining family conflict as a mechanism of change. *Journal of Family Psychology : JFP : Journal of the Division of Family Psychology of the American Psychological Association (Division 43)*, *30*(1), 82–92. https://doi.org/10.1037/fam0000147

Gladstone, T., Marko-Holguin, M., Henry, J., Fogel, J., Diehl, A., & van Voorhees, B. W. (2014). Understanding adolescent response to a technology-based depression prevention program. *Journal of Clinical Child and Adolescent Psychology : The Official Journal for the Society of Clinical Child and Adolescent Psychology, American Psychological Association, Division 53*, *43*(1), 102–114. https://doi.org/10.1080/15374416.2013.850697

Goldstein, T. R., Krantz, M. L., Fersch-Podrat, R. K., Hotkowski, N. J., Merranko, J., Sobel, L., Axelson, D., Birmaher, B [Boris], & Douaihy, A. (2020). A brief motivational intervention for enhancing medication adherence for adolescents with bipolar disorder: A pilot randomized trial. *Journal of Affective Disorders*, *265*, 1–9. https://doi.org/10.1016/j.jad.2020.01.015

Gonzales, N. A., Dumka, L. E., Millsap, R. E., Gottschall, A., McClain, D. B., Wong, J. J., Germán, M., Mauricio, A. M., Wheeler, L., Carpentier, F. D., & Kim, S. Y. (2012). Randomized trial of a broad preventive intervention for Mexican American adolescents. *Journal of Consulting and Clinical Psychology*, *80*(1), 1–16. https://doi.org/10.1037/a0026063

Harrington, R., Kerfoot, M., Dyer, E., Mcniven, F., Gill, J., Harrington, V., & Woodham, A. (2000). Deliberate self-poisoning in adolescence: Why does a brief family intervention work in some cases and not others? *Journal of Adolescence*, *23*(1), 13–20. https://doi.org/10.1006/jado.1999.0293

Henggeler, S. W [Scott W.], Letourneau, E. J., Chapman, J. E., Borduin, C. M., Schewe, P. A., & McCart, M. R. (2009). Mediators of change for multisystemic therapy with juvenile sexual offenders. *Journal of Consulting and Clinical Psychology*, *77*(3), 451–462. https://doi.org/10.1037/a0013971

Henggeler, S. W [S. W.], Melton, G. B., & Smith, L. A. (1992). Family preservation using multisystemic therapy: An effective alternative to incarcerating serious juvenile offenders. *Journal of Consulting and Clinical Psychology*, *60*(6), 953–961. https://doi.org/10.1037//0022-006x.60.6.953

Hogendoorn, S. M [Sanne M.], Prins, P. J. M [Pier J. M.], Boer, F [Frits], Vervoort, L [Leentje], Wolters, L. H [Lidewij H.], Moorlag, H., Nauta, M. H., Garst, H., Hartman, C. A., & Haan, E. de [Else] (2014). Mediators of cognitive behavioral therapy for anxiety-disordered children and adolescents: Cognition, perceived control, and coping. *Journal of Clinical Child and Adolescent Psychology : The Official Journal for the Society of Clinical Child and Adolescent Psychology, American Psychological Association, Division 53*, *43*(3), 486–500. https://doi.org/10.1080/15374416.2013.807736

Hogue, A., Dauber, S., Stambaugh, L. F., Cecero, J. J., & Liddle, H. A. (2006). Early therapeutic alliance and treatment outcome in individual and family therapy for adolescent behavior problems. *Journal of Consulting and Clinical Psychology*, *74*(1), 121–129. https://doi.org/10.1037/0022-006X.74.1.121

Huey, S. J., Henggeler, S. W [S. W.], Brondino, M. J., & Pickrel, S. G. (2000). Mechanisms of change in multisystemic therapy: Reducing delinquent behavior through therapist adherence and improved family and peer functioning. *Journal of Consulting and Clinical Psychology*, *68*(3), 451–467.

Jacobs, R. H., Silva, S. G., Reinecke, M. A., Curry, J. F., Ginsburg, G. S., Kratochvil, C. J., & March, J. S. (2009). Dysfunctional attitudes scale perfectionism: A predictor and partial mediator of acute treatment outcome among clinically depressed adolescents. *Journal of Clinical Child and Adolescent Psychology : The Official Journal for the Society of Clinical Child and Adolescent Psychology, American Psychological Association, Division 53*, *38*(6), 803–813. https://doi.org/10.1080/15374410903259031

Jensen, T. K., Holt, T., Mørup Ormhaug, S., Fjermestad, K. W., & Wentzel-Larsen, T. (2018). Change in post-traumatic cognitions mediates treatment effects for traumatized youth-A randomized controlled trial. *Journal of Counseling Psychology*, *65*(2), 166–177. https://doi.org/10.1037/cou0000258

Jones, J. D., Gallop, R., Gillham, J. E., Mufson, L., Farley, A. M., Kanine, R., & Young, J. F. (2021). The Depression Prevention Initiative: Mediators of Interpersonal Psychotherapy-Adolescent Skills Training. *Journal of Clinical Child and Adolescent Psychology : The Official Journal for the Society of Clinical Child and Adolescent Psychology, American Psychological Association, Division 53*, *50*(2), 202–214. https://doi.org/10.1080/15374416.2019.1644648

Kangaslampi, S., Punamäki, R.‑L., Qouta, S., Diab, M., & Peltonen, K. (2016). Psychosocial Group Intervention Among War-Affected Children: An Analysis of Changes in Posttraumatic Cognitions. *Journal of Traumatic Stress*, *29*(6), 546–555. https://doi.org/10.1002/jts.22149

Kashikar-Zuck, S., Sil, S., Lynch-Jordan, A. M., Ting, T. V., Peugh, J., Schikler, K. N., Hashkes, P. J., Arnold, L. M., Passo, M., Richards-Mauze, M. M., Powers, S. W., & Lovell, D. J. (2013). Changes in pain coping, catastrophizing, and coping efficacy after cognitive-behavioral therapy in children and adolescents with juvenile fibromyalgia. *The Journal of Pain*, *14*(5), 492–501. https://doi.org/10.1016/j.jpain.2012.12.019

Kauer, S. D., Reid, S. C., Crooke, A. H. D., Khor, A., Hearps, S. J. C., Jorm, A. F [Anthony Francis], Sanci, L., & Patton, G. (2012). Self-monitoring using mobile phones in the early stages of adolescent depression: Randomized controlled trial. *Journal of Medical Internet Research*, *14*(3), e67. https://doi.org/10.2196/jmir.1858

Kaufman, N. K., Rohde, P., Seeley, J. R., Clarke, G. N., & Stice, E. (2005). Potential mediators of cognitive-behavioral therapy for adolescents with comorbid major depression and conduct disorder. *Journal of Consulting and Clinical Psychology*, *73*(1), 38–46. https://doi.org/10.1037/0022-006X.73.1.38

Kenney, S. R., Napper, L. E., LaBrie, J. W., & Martens, M. P. (2014). Examining the efficacy of a brief group protective behavioral strategies skills training alcohol intervention with college women. *Psychology of Addictive Behaviors*, *28*(4), 1041–1051. https://doi.org/10.1037/a0038173

Knutsen, M. L., Czajkowski, N. O., & Ormhaug, S. M. (2018). Changes in posttraumatic stress symptoms, cognitions, and depression during treatment of traumatized youth. *Behaviour Research and Therapy*, *111*, 119–126. https://doi.org/10.1016/j.brat.2018.10.010

Kwok, S. Y. C. L. (2019). Integrating Positive Psychology and Elements of Music Therapy to Alleviate Adolescent Anxiety. *Research on Social Work Practice*, *29*(6), 663–676. https://doi.org/10.1177/1049731518773423

Le Grange, D., Lock, J., Agras, W. S., Moye, A., Bryson, S. W., Jo, B., & Kraemer, H. C. (2012). Moderators and mediators of remission in family-based treatment and adolescent focused therapy for anorexia nervosa. *Behaviour Research and Therapy*, *50*(2), 85–92. https://doi.org/10.1016/j.brat.2011.11.003

Lewis, C. C., Simons, A. D., Silva, S. G., Rohde, P., Small, D. M., Murakami, J. L., High, R. R., & March, J. S. (2009). The role of readiness to change in response to treatment of adolescent depression. *Journal of Consulting and Clinical Psychology*, *77*(3), 422–428. https://doi.org/10.1037/a0014154

Lindsey, M. A., Romanelli, M., Ellis, M. L., Barker, E. D., Boxmeyer, C. L., & Lochman, J. E. (2019). The Influence of Treatment Engagement on Positive Outcomes in the Context of a School-Based Intervention for Students with Externalizing Behavior Problems. *Journal of Abnormal Child Psychology*, *47*(9), 1437–1454. https://doi.org/10.1007/s10802-019-00525-6

Magill, M., Colby, S. M., Orchowski, L., Murphy, J. G., Hoadley, A., Brazil, L. A., & Barnett, N. P. (2017). How does brief motivational intervention change heavy drinking and harm among underage young adult drinkers? *Journal of Consulting and Clinical Psychology*, *85*(5), 447–458. https://doi.org/10.1037/ccp0000200

Magill, M., Janssen, T., Mastroleo, N., Hoadley, A., Walthers, J., Barnett, N., & Colby, S. (2019). Motivational interviewing technical process and moderated relational process with underage young adult heavy drinkers. *Psychology of Addictive Behaviors*, *33*(2), 128–138. https://doi.org/10.1037/adb0000440

McLean, C. P., Yeh, R., Rosenfield, D., & Foa, E. B. (2015). Changes in negative cognitions mediate PTSD symptom reductions during client-centered therapy and prolonged exposure for adolescents. *Behaviour Research and Therapy*, *68*, 64–69. https://doi.org/10.1016/j.brat.2015.03.008

McNally, A. M., Palfai, T. P., & Kahler, C. W. (2005). Motivational interventions for heavy drinking college students: Examining the role of discrepancy-related psychological processes. *Psychology of Addictive Behaviors*, *19*(1), 79–87. https://doi.org/10.1037/0893-164X.19.1.79

Mehlum, L., Ramleth, R.‑K., Tørmoen, A. J., Haga, E., Diep, L. M., Stanley, B. H., Miller, A. L., Larsson, B., Sund, A. M., & Grøholt, B. (2019). Long term effectiveness of dialectical behavior therapy versus enhanced usual care for adolescents with self-harming and suicidal behavior. *Journal of Child Psychology and Psychiatry, and Allied Disciplines*, *60*(10), 1112–1122. https://doi.org/10.1111/jcpp.13077

Mehlum, L., Tørmoen, A. J., Ramberg, M., Haga, E., Diep, L. M., Laberg, S., Larsson, B. S., Stanley, B. H., Miller, A. L., Sund, A. M., & Grøholt, B. (2014). Dialectical behavior therapy for adolescents with repeated suicidal and self-harming behavior: A randomized trial. *Journal of the American Academy of Child and Adolescent Psychiatry*, *53*(10), 1082–1091. https://doi.org/10.1016/j.jaac.2014.07.003

Meiser-Stedman, R., Smith, P., McKinnon, A., Dixon, C., Trickey, D., Ehlers, A., Clark, D. M., Boyle, A., Watson, P., Goodyer, I., & Dalgleish, T. (2017). Cognitive therapy as an early treatment for post-traumatic stress disorder in children and adolescents: A randomized controlled trial addressing preliminary efficacy and mechanisms of action. *Journal of Child Psychology and Psychiatry, and Allied Disciplines*, *58*(5), 623–633. https://doi.org/10.1111/jcpp.12673

Murphy, J. G., Dennhardt, A. A., Skidmore, J. R., Borsari, B [Brian], Barnett, N. P., Colby, S. M., & Martens, M. P. (2012). A randomized controlled trial of a behavioral economic supplement to brief motivational interventions for college drinking. *Journal of Consulting and Clinical Psychology*, *80*(5), 876–886. https://doi.org/10.1037/a0028763

Murphy, J. G., Dennhardt, A. A., Martens, M. P., Borsari, B [Brian], Witkiewitz, K., & Meshesha, L. Z. (2019). A randomized clinical trial evaluating the efficacy of a brief alcohol intervention supplemented with a substance-free activity session or relaxation training. *Journal of Consulting and Clinical Psychology*, *87*(7), 657–669. https://doi.org/10.1037/ccp0000412

Norr, A. M., Allan, N. P., Macatee, R. J., Keough, M. E., & Schmidt, N. B. (2014). The effects of an anxiety sensitivity intervention on anxiety, depression, and worry: Mediation through affect tolerances. *Behaviour Research and Therapy*, *59*, 12–19. https://doi.org/10.1016/j.brat.2014.05.011

Norr, A. M., Gibby, B. A., & Schmidt, N. B. (2017). Is computerized psychoeducation sufficient to reduce anxiety sensitivity in an at-risk sample? A randomized trial. *Journal of Affective Disorders*, *212*, 48–55. https://doi.org/10.1016/j.jad.2017.01.032

O'Leary-Barrett, M., Pihl, R. O., & Conrod, P. J. (2017). Process variables predicting changes in adolescent alcohol consumption and mental health symptoms following personality-targeted interventions. *Addictive Behaviors*, *75*, 47–58. https://doi.org/10.1016/j.addbeh.2017.06.022

Ollendick, T. H., Ryan, S. M., Capriola-Hall, N. N., Reuterskiöld, L., & Öst, L.‑G. (2017). The mediating role of changes in harm beliefs and coping efficacy in youth with specific phobias. *Behaviour Research and Therapy*, *99*, 131–137. https://doi.org/10.1016/j.brat.2017.10.007

Orkibi, H., Azoulay, B., Snir, S., & Regev, D. (2017). In-session behaviours and adolescents' self-concept and loneliness: A psychodrama process-outcome study. *Clinical Psychology & Psychotherapy*, *24*(6), O1455-O1463. https://doi.org/10.1002/cpp.2103

Orlando, M., Ellickson, P. L., McCaffrey, D. F., & Longshore, D. L. (2005). Mediation analysis of a school-based drug prevention program: Effects of Project ALERT. *Prevention Science : The Official Journal of the Society for Prevention Research*, *6*(1), 35–46. https://doi.org/10.1007/s11121-005-1251-z

Pantin, H., Prado, G., Lopez, B., Huang, S., Tapia, M. I., Schwartz, S. J., Sabillon, E., Brown, C. H., & Branchini, J. (2009). A randomized controlled trial of Familias Unidas for Hispanic adolescents with behavior problems. *Psychosomatic Medicine*, *71*(9), 987–995. https://doi.org/10.1097/PSY.0b013e3181bb2913

Paquette, J., & Vitaro, F. (2014). Wilderness Therapy, Interpersonal Skills and Accomplishment Motivation: Impact Analysis on Antisocial Behavior and Socio-Professional Status. *Residential Treatment for Children & Youth*, *31*(3), 230–252. https://doi.org/10.1080/0886571X.2014.944024

Peris, T. S., Rozenman, M. S., Sugar, C. A., McCracken, J. T., & Piacentini, J. (2017). Targeted Family Intervention for Complex Cases of Pediatric Obsessive-Compulsive Disorder: A Randomized Controlled Trial. *Journal of the American Academy of Child and Adolescent Psychiatry*, *56*(12), 1034-1042.e1. https://doi.org/10.1016/j.jaac.2017.10.008

Perrino, T., Pantin, H., Huang, S., Brincks, A., Brown, C. H., & Prado, G. (2016). Reducing the Risk of Internalizing Symptoms among High-risk Hispanic Youth through a Family Intervention: A Randomized Controlled Trial. *Family Process*, *55*(1), 91–106. https://doi.org/10.1111/famp.12132

Pfeiffer, E., Sachser, C., Haan, A. de, Tutus, D., & Goldbeck, L. (2017). Dysfunctional posttraumatic cognitions as a mediator of symptom reduction in Trauma-Focused Cognitive Behavioral Therapy with children and adolescents: Results of a randomized controlled trial. *Behaviour Research and Therapy*, *97*, 178–182. https://doi.org/10.1016/j.brat.2017.08.001

Pineda, J., & Dadds, M. R [Mark R.] (2013). Family intervention for adolescents with suicidal behavior: A randomized controlled trial and mediation analysis. *Journal of the American Academy of Child and Adolescent Psychiatry*, *52*(8), 851–862. https://doi.org/10.1016/j.jaac.2013.05.015

Riley, A. R., Duke, D. C., Freeman, K. A., Hood, K. K., & Harris, M. A. (2015). Depressive Symptoms in a Trial Behavioral Family Systems Therapy for Diabetes: A Post Hoc Analysis of Change. *Diabetes Care*, *38*(8), 1435–1440. https://doi.org/10.2337/dc14-2519

Rossouw, T. I., & Fonagy, P. (2012). Mentalization-based treatment for self-harm in adolescents: A randomized controlled trial. *Journal of the American Academy of Child and Adolescent Psychiatry*, *51*(12), 1304-1313.e3. https://doi.org/10.1016/j.jaac.2012.09.018

Schleider, J. L., Ginsburg, G. S., Keeton, C. P., Weisz, J. R., Birmaher, B [Boris], Kendall, P. C [Phillip C.], Piacentini, J., Sherrill, J., & Walkup, J. T. (2015). Parental psychopathology and treatment outcome for anxious youth: Roles of family functioning and caregiver strain. *Journal of Consulting and Clinical Psychology*, *83*(1), 213–224. https://doi.org/10.1037/a0037935

Seidel, A., Presnell, K., & Rosenfield, D. (2009). Mediators in the dissonance eating disorder prevention program. *Behaviour Research and Therapy*, *47*(8), 645–653. https://doi.org/10.1016/j.brat.2009.04.007

Smith, P., Scott, R., Eshkevari, E., Jatta, F., Leigh, E., Harris, V., Robinson, A., Abeles, P., Proudfoot, J., Verduyn, C., & Yule, W. (2015). Computerised CBT for depressed adolescents: Randomised controlled trial. *Behaviour Research and Therapy*, *73*, 104–110. https://doi.org/10.1016/j.brat.2015.07.009

Smith, P., Yule, W., Perrin, S., Tranah, T., Dalgleish, T., & Clark, D. M. (2007). Cognitive-behavioral therapy for PTSD in children and adolescents: A preliminary randomized controlled trial. *Journal of the American Academy of Child and Adolescent Psychiatry*, *46*(8), 1051–1061. https://doi.org/10.1097/CHI.0b013e318067e288

Smits, J. A. J., Rosenfield, D., McDonald, R., & Telch, M. J. (2006). Cognitive mechanisms of social anxiety reduction: An examination of specificity and temporality. *Journal of Consulting and Clinical Psychology*, *74*(6), 1203–1212. https://doi.org/10.1037/0022-006X.74.6.1203

Stice, E., Presnell, K., Gau, J., & Shaw, H. (2007). Testing mediators of intervention effects in randomized controlled trials: An evaluation of two eating disorder prevention programs. *Journal of Consulting and Clinical Psychology*, *75*(1), 20–32. https://doi.org/10.1037/0022-006X.75.1.20

Stice, E., Rohde, P., Seeley, J. R., & Gau, J. M. (2010). Testing mediators of intervention effects in randomized controlled trials: An evaluation of three depression prevention programs. *Journal of Consulting and Clinical Psychology*, *78*(2), 273–280. https://doi.org/10.1037/a0018396

Stice, E., Rohde, P., Gau, J., & Ochner, C. (2011). Relation of depression to perceived social support: Results from a randomized adolescent depression prevention trial. *Behaviour Research and Therapy*, *49*(5), 361–366. https://doi.org/10.1016/j.brat.2011.02.009

Summers, B. J., & Cougle, J. R. (2016). Modifying interpretation biases in body dysmorphic disorder: Evaluation of a brief computerized treatment. *Behaviour Research and Therapy*, *87*, 117–127. https://doi.org/10.1016/j.brat.2016.09.005

Swain, J., Hancock, K., Hainsworth, C., & Bowman, J. (2015). Mechanisms of change: Exploratory outcomes from a randomised controlled trial of acceptance and commitment therapy for anxious adolescents. *Journal of Contextual Behavioral Science*, *4*(1), 56–67. https://doi.org/10.1016/j.jcbs.2014.09.001

Tan, L., & Martin, G. (2015). Taming the adolescent mind: A randomised controlled trial examining clinical efficacy of an adolescent mindfulness-based group programme. *Child and Adolescent Mental Health*, *20*(1), 49–55. https://doi.org/10.1111/camh.12057

Tein, J.‑Y., Sandler, I. N., Ayers, T. S., & Wolchik, S. A. (2006). Mediation of the effects of the family bereavement program on mental health problems of bereaved children and adolescents. *Prevention Science : The Official Journal of the Society for Prevention Research*, *7*(2), 179–195. https://doi.org/10.1007/s11121-006-0037-2

Timpano, K. R., Raines, A. M., Shaw, A. M., Keough, M. E., & Schmidt, N. B. (2016). Effects of a brief anxiety sensitivity reduction intervention on obsessive compulsive spectrum symptoms in a young adult sample. *Journal of Psychiatric Research*, *83*, 8–15. https://doi.org/10.1016/j.jpsychires.2016.07.022

Topper, M., Emmelkamp, P. M. G., Watkins, E., & Ehring, T. (2017). Prevention of anxiety disorders and depression by targeting excessive worry and rumination in adolescents and young adults: A randomized controlled trial. *Behaviour Research and Therapy*, *90*, 123–136. https://doi.org/10.1016/j.brat.2016.12.015

Tutus, D., Goldbeck, L., Pfeiffer, E., Sachser, C., & Plener, P. L. (2019). Parental dysfunctional posttraumatic cognitions in trauma-focused cognitive behavioral therapy for children and adolescents. *Psychological Trauma : Theory, Research, Practice and Policy*, *11*(7), 722–731. https://doi.org/10.1037/tra0000419

van Ryzin, M. J., & Leve, L. D. (2012). Affiliation with delinquent peers as a mediator of the effects of multidimensional treatment foster care for delinquent girls. *Journal of Consulting and Clinical Psychology*, *80*(4), 588–596. https://doi.org/10.1037/a0027336

Weintraub, M., Schneck, C., Posta, F., Merranko, J., Singh, M., Chang, K., & Miklowitz, D. (2022). Effects of family intervention on psychosocial functioning and mood symptoms of youth at high risk for bipolar disorder. *Journal Of Consulting And Clinical Psychology*, *90*(2), 161-171. doi: 10.1037/ccp0000708

Werch, C. E., Bian, H., Carlson, J. M., Moore, M. J., Diclemente, C. C., Huang, I.‑C., Ames, S. C., Thombs, D., Weiler, R. M., & Pokorny, S. B. (2011). Brief integrative multiple behavior intervention effects and mediators for adolescents. *Journal of Behavioral Medicine*, *34*(1), 3–12. https://doi.org/10.1007/s10865-010-9281-9

Winters, K. C., Fahnhorst, T., Botzet, A., Lee, S., & Lalone, B. (2012). Brief intervention for drug-abusing adolescents in a school setting: Outcomes and mediating factors. *Journal of Substance Abuse Treatment*, *42*(3), 279–288. https://doi.org/10.1016/j.jsat.2011.08.005

Winters, K. C., Lee, S., Botzet, A., Fahnhorst, T., & Nicholson, A. (2014). One-year outcomes and mediators of a brief intervention for drug abusing adolescents. *Psychology of Addictive Behaviors : Journal of the Society of Psychologists in Addictive Behaviors*, *28*(2), 464–474. https://doi.org/10.1037/a0035041

Wolters, L. H [L. H.], Prins, P. J. M [P. J. M.], Garst, G. J. A., Hogendoorn, S. M [S. M.], Boer, F [F.], Vervoort, L [L.], & Haan, E. de [E.] (2019). Mediating Mechanisms in Cognitive Behavioral Therapy for Childhood OCD: The Role of Dysfunctional Beliefs. *Child Psychiatry and Human Development*, *50*(2), 173–185. https://doi.org/10.1007/s10578-018-0830-8

Wu, M. S., Caporino, N. E., Peris, T. S., Pérez, J., Thamrin, H., Albano, A. M., Kendall, P. C [Philip C.], Walkup, J. T., Birmaher, B [Boris], Compton, S. N., & Piacentini, J. (2020). The Impact of Treatment Expectations on Exposure Process and Treatment Outcome in Childhood Anxiety Disorders. *Journal of Abnormal Child Psychology*, *48*(1), 79–89. https://doi.org/10.1007/s10802-019-00574-x

Yap, M. B. H., Cardamone-Breen, M. C., Rapee, R. M., Lawrence, K. A., Mackinnon, A. J., Mahtani, S., & Jorm, A. F [Anthony F.] (2019). Medium-Term Effects of a Tailored Web-Based Parenting Intervention to Reduce Adolescent Risk of Depression and Anxiety: 12-Month Findings From a Randomized Controlled Trial. *Journal of Medical Internet Research*, *21*(8), e13628. https://doi.org/10.2196/13628

Zhou, Y., Arend, J., Mufson, L., & Gunlicks-Stoessel, M. (2021). Change in dysfunctional attitudes and attachment in interpersonal psychotherapy for depressed adolescents. *Psychotherapy Research : Journal of the Society for Psychotherapy Research*, *31*(2), 258–266. https://doi.org/10.1080/10503307.2020.1756513
